# Effects of hypoxia stress on the milk synthesis in bovine mammary epithelial cells

**DOI:** 10.1186/s40104-025-01174-0

**Published:** 2025-03-07

**Authors:** Yanshan Jin, Zhuolin Liu, Ziyan Yang, Lizhu Fang, Feng-Qi Zhao, Hongyun Liu

**Affiliations:** 1 College of Animal Sciences, Zhejiang University, Hangzhou, 310058 China; 2 Department of Animal and Veterinary Sciences, University of Vermont, Burlington, VT 05405 USA

**Keywords:** Hypoxic stress, HIF-1α, Low oxygen, Milk synthesis

## Abstract

**Background:**

Milk synthesis is an energy-intensive process influenced by oxygen availability. This study investigates how hypoxia affects milk synthesis in BMECs, focusing on key genes involved in lactation and energy metabolism.

**Methods:**

BMECs were cultured in a normoxic environment and then transferred to a hypoxia chamber with 1% O_2_ for specified durations. The study evaluated cellular responses through various molecular experiments and RNA sequencing. Small interfering RNA was employed to knock down *HIF-1α* to investigate whether the lactation-related phenotype alteration depends on HIF-1α.

**Results:**

Hypoxia disrupted milk protein production by reducing mTOR/P70S6K/4EBP1 signaling and downregulating genes critical for amino acid transport and protein synthesis. Triglyceride synthesis increased due to enhanced fatty acid uptake and the upregulation of regulatory proteins, including FASN and PPARγ. Although glucose uptake was elevated under hypoxia, key enzymes for lactose synthesis were downregulated, suggesting a redirection of glucose toward energy production. Mitochondrial function was impaired under hypoxia, with reduced gene expression in TCA cycle, ETC, cytosol-mitochondrial transport, decreased ATP levels, increased ROS levels, and structural alterations. Additionally, lipid synthesis and glucose uptake depend on HIF-1α, while milk protein synthesis alterations occurred independently of HIF-1α.

**Conclusions:**

Hypoxia alters milk synthesis in BMECs by disrupting milk protein synthesis, enhancing lipid metabolism, and impairing energy production. These findings provide valuable insights into the molecular mechanisms underlying the effect of oxygen deprivation on lactation efficiency, offering potential targets for mitigating hypoxic stress in the mammary glands of dairy animals.

**Supplementary Information:**

The online version contains supplementary material available at 10.1186/s40104-025-01174-0.

## Background

Oxygen is the final electron receptor of oxidative phosphorylation in ATP production, which is the primary mode of energy synthesis in advanced organisms. Recent studies found that lactation persistency is highly correlated with oxygen availability since milk synthesis is significantly energy demanded thus effective oxygen usage and energy production are of importance [1]. The effectiveness of aerobic oxidation is determined by the availability of oxygen [2]. In most tissues and organs of the body, the partial pressure of oxygen ranges from 10 to 45 mm Hg [3, 4]. Hypoxia refers to the physiological state in which the oxygen level in a tissue or organ is insufficient to support the body’s regular metabolic activities [3]. Although most tissues and organs have different oxygen levels, an oxygen concentration of 6% is enough to initiate the hypoxic response in most tissues, and an oxygen concentration of 0.5%–1% can produce severe hypoxia in most tissues [5, 6]. Disrupted oxygen homeostasis can result in decreased efficiency in energy production and accumulation of hazardous by products.

Hypoxia-inducible factor-1 (HIF-1) is a fundamental regulator of the tissues to react to oxygen levels [7]. HIF-1 is a heterodimer complex made up of α and β subunits. While the expression of the β subunit is mostly confined in the nucleus and is generally constant, the expression of the α subunit is controlled by oxygen levels [8, 9]. Proline hydroxylase domains (PHDs) and the Von Hippel-Lindau protein (VHL) under normoxic circumstances hydroxylate HIF-1 before its ubiquitination [9]. Hydroxylated HIF-1 has a diminished nuclear translocation activity and will be captured by the proteasome for degradation [10, 11]. HIF-1α hydroxylation is hindered under hypoxic settings, causing HIF-1 to accumulate and translocate to the nucleus where it combines with HIF-1β to generate a dimer bind to the hypoxia response elements of the target genes [12, 13].

Hypoxia has been associated with mammary morphogenesis, secretory differentiation, lactation initiation and lactation persistency in previous studies [14–19]. However, its specific effects on milk synthesis remain largely unexplored. Therefore, the primary aim of this study is to examine the influence of hypoxia on the milk synthesis capacity of bovine mammary epithelial cells (BMECs). Additionally, the secondary aims of this study are to 1) investigate the effects of hypoxia on the key lactation-related pathways in BMECs; 2) elucidate hypoxia-induced transcriptomic changes of the genes related to lactation and energy metabolism; and 3) assess the role of HIF-1α in regulating lactation capacity in BMECs under hypoxic conditions.

## Materials and methods

### Cell culture

The procedures of BMECs culture were described previously [20]. The mammary gland tissues were obtained from 3 mid-lactation Holstein dairy cows. The use of animal tissues was approved by the Institutional Animal Use Committee of Zhejiang University. The cells were cultured in a complete medium [Dulbecco's modified Eagle's medium (DMEM)-F12 supplemented with 5 μg/mL transferrin (T0665, Sigma), 5 μg/mL insulin (I8040, Solarbio, Beijing, China), 1 μg/mL hydrocortisone (G8450, Solarbio), 10 ng/mL epidermal growth factor (E4127, Sigma), 1% (w/v) penicillin−streptomycin (CP011, Keyi), and 10% (w/v) fetal bovine serum (FBS, 10099141, Gibco)] in a humidified atmosphere containing 5% (v/v) CO_2_.

For hypoxia treatment, BMECs were cultivated in a normal environment [37 °C , 5% (v/v) CO_2_, 95% (v/v) air] for 24 h prior to hypoxia treatment. After replacing with fresh culture medium, BMECs were moved to a hypoxia chamber (Billups-rothenberg, Del Mar, CA, USA) that contained a mixed hypoxia gas [37 °C , 1% (v/v) O_2_, 5% (v/v) CO_2_, and 94% (v/v) N_2_] for varying periods of time (0, 2, 4, 6, 12, 24, and 36 h). Phosphate-buffered saline (PBS) was used to maintain a humidified atmosphere in the hypoxia chamber. Cells constantly cultured in normoxia were used as corresponding controls. A detailed scheme of study design was shown in Fig. S1.

### Small interfering RNA transfection

After reaching 40% confluence, BMECs were transfected with a negative control (NC) siRNA (GenePharma, Shanghai, China) or a custom-designed *HIF-1*α siRNA (GenePharma) with Lipofectamine™ RNAiMAX Transfection Reagent (13778150, Invitrogen, Waltham, MA, USA). The *HIF-1*α siRNA were 5′-GCCGCUCAAUUUAUGAAUATT-3′ (forward) and 5′-UAUUCAUAAAUUGAGCGGCTT-3′ (reverse). The negative control (NC) siRNA was 5′-UUCUCCGAACGUGUCACGUTT-3′ (forward) and 5′-ACGUGACACGUUCGGAGAATT-3′ (reverse). After 6 h of transfection, the medium was replaced with complete medium, and the cells were cultured for an additional 24 h. Following this, the medium was refreshed with fresh complete medium. For normoxia or hypoxia treatments, the cells were incubated under normoxic or hypoxic conditions at 37 °C for another 24 h.

### Immunoblot

BMECs were lysed in ice-cold RIPA buffer (P0013B, Beyotime, Shanghai, China) supplemented with 1 × protease phosphatase inhibitor mixture (P1045, Beyotime). The cell lysates were collected after centrifugation at 10,000 × *g* for 10 min at 4 °C. A BCA protein concentration assay kit (P0009, Beyotime) was used to measure the protein concentration of the cell lysates before adjusting their protein concentrations to 1 mg/mL for each sample. Equivalent amounts of 10 μg total proteins of cell lysates were separated by sodium dodecyl sulfate (SDS)-polyacrylamide gel electrophoresis (PAGE) and electro-transferred to polyvinylidene difluoride membranes (IPVH00010, Sigma). The membranes were probed with primary antibodies and HRP-conjugated secondary antibodies (Affinity, Liyang, Jiangsu, China). The antibodies used for this study were listed in Table S1. The images were captured by a chemiluminescence system (CLiNX Science Instrument, Shanghai, China). Images were analyzed by the ImageJ software (National Institutes of Health, Bethesda, MD, USA). The relative protein levels were normalized by the levels of β-actin.

### Lipid droplet staining

BMECs were seeded into laser confocal dishes (NEST, Wuxi, Jiangsu, China). BMECs were washed with sterile PBS, and fixed with 10% (w/v) neutral buffered formaldehyde at room temperature for 10 min. Following that, BMECs were stained with BODIPY 493/503 (D3922, Invitrogen) for 15 min and DAPI (G1012, Servicebio, Wuhan, China) for 1 min at room temperature. The final staining images were observed and captured with an Olympus IX81-FV1000 laser confocal microscope (Olympus, Tokyo, Japan). Images were analyzed by the ImageJ software (National Institutes of Health).

### Glucose uptake assay

The cell culture supernatant was collected and centrifuged at 4 °C for 10 min. The glucose concentration was detected with an automatic chemistry analyzer (7020, Hitachi, Tokyo, Japan). The original glucose concentration in the culture medium (17.51 mmol/L) was subtracted from the measured glucose concentration, and the resulting value was divided by the sample’s protein concentration to correct for cell number differences.

### Triglyceride content measurement

The triglyceride content was measured using a commercial kit (E1013, Applygen, Beijing, China). Briefly, BMECs were lysed using the lysis buffer in the kit. The BMECs lysate was centrifuged at 12,000 × *g* at 4 °C for 5 min, and the supernatant was collected. A portion of the supernatant was used to measure the protein concentration using a BCA protein concentration kit. The residual supernatant was heated in a water bath at 70 °C for 10 min, centrifuged at 2000 r/min for 5 min, and then collected again. A 10 μL supernatant was mixed with 190 μL of chromogenic liquid before triglyceride content was measured at 550 nm absorbance. To adjust for variances in cell number, the triglyceride content was divided by the protein concentration.

### ATP content assay

BMECs were seeded onto opaque wall perforated 96-well plates and cultured. After hypoxic or normoxic treatments, the cultured cells were placed to room temperature for 30 min, the cellular ATP content was detected with CellTiter-Glo® 2.0 assay kit (G9241, Promega, Beijing, China). The 100 μL CellTiter-Glo® 2.0 Reagent was added to each well and shaken for 2 min to induce cell lysis. The ATP standard curve was made using ATP disodium salt (P1132, Promega, Beijing, China) diluted by DMEM-F12. After incubated at room temperature for 10 min, the chemiluminescence values were recorded using a microplate reader (Tecan, San Jose, CA, USA).

### Flow cytometry

For the mitochondrial mass detection, mito-tracker green (C1048, Beyotime) was used to mark mitochondria. BMECs were seeded into 6-well plates and underwent hypoxic or normoxic treatments. Cells were collected in 1.5-mL centrifuge tubes. After washing the cells with sterile PBS, BMECs were re-suspended in the mito-tracker green working solution (1:10,000 diluted in HBSS solution) and incubated at 37 °C for 30 min. A flow cytometer (BD Biosciences, Franklin Lakes, NJ, USA) was used to measure the fluorescence density at excitation/emission = 490/516 nm.

### Transmission electron microscopy

BMECs were harvested and then fixed with 2.5% (v/v) glutaraldehyde at 4 °C for 12 h. After washing with PBS, samples were fixed in 1% (w/v) osmic acid at room temperature for 1 h. The samples were rinsed with distilled water and stained for 30 min with a 2% (w/v) uranium acetate aqueous solution. Samples were then dehydrated in a series of increasing concentrations of alcohol before being embedded in an embedding medium. After ultrathin sectioning, BMECs were observed using a Talos L120C transmission electron microscope (ThermoFisher Scientific, Waltham, MA, USA).

### RNA sequencing

Total RNA was extracted from cells using Trizol reagent (Invitrogen) following the manufacturer’s procedures. The total RNA quantity and purity were analyzed using Bioanalyzer 2100 and RNA 6000 Nano LabChip Kit (Agilent, Santa Clara, CA, USA) with RIN number > 7.0. A library with a fragment size of 300 bp ± 50 bp was formed from the pooled RNA. cDNA library was pair-ended sequenced using Illumina Novaseq™ 6000 (LC-Bio Technology, Hangzhou, China) in PE150 sequencing mode according to standard procedures. Fastp software (https://github.com/OpenGene/fastp) was used to remove the reads that contained adaptor contamination, low-quality bases, and undetermined bases with default parameters. StringTie (https://ccb.jhu.edu/software/stringtie) was used to determine the expression level for mRNAs by calculating FPKM (FPKM = [total_exon_fragments/mapped_reads (millions) × exon_length (kB)]). Differential expression analysis was performed using the R package edgeR (https://bioconductor.org/packages/release/bioc/html/edgeR.html). Genes with a fold-change ≥ 2 and a *P*-value < 0.05 were assigned as differential expressed genes (DEGs). Gene ontology (GO) enrichment analyses were performed using the OmicStudio tools (https://www.omicstudio.cn/tool). We hypothesize that the expression of key genes involved in milk protein, fat, and lactose synthesis will change significantly in the HP6 vs. HP0 and HP24 vs. HP0 comparisons. Additionally, genes associated with energy metabolism and mitochondrial function are expected to exhibit significant expression changes in these comparisons. These key lactation-related genes were identified based on previous studies [21–26]. Genes related to energy metabolism were selected based on GO terms: GO:0006096 glycolytic process, GO:0006099 (tricarboxylic acid (TCA) cycle), GO:0006119 (oxidative phosphorylation), and GO:0030150 (protein import into mitochondrial matrix), using the QuickGO database (https://www.ebi.ac.uk/QuickGO/) provided by the European Molecular Biology Laboratory—European Bioinformatics Institute (EMBL-EBI) [27]. The genes related to mitochondrial function (i.e., mitophagy, autophagy and reactive oxygen species (ROS) production) were selected from previous studies [28–30].

### Transcription factor enrichment analysis

DEGs from the HP24 vs. HP0 comparison were analyzed to identify enriched transcription factors using the web-based ChIP-X Enrichment Analysis (ChEA3) version 3 platform (https://maayanlab.cloud/chea3/). A total of 939 DEGs were initially submitted for the query. After excluding genes with unrecognized IDs, 736 DEGs were included in the final analysis. The "Top Rank" library from the ChEA3 database was utilized for this analysis. Overlapping genes were identified by comparing the input gene set with the target gene set list from the ChEA3 database. The results from the ChEA3 transcription factor target libraries are ranked based on Fisher's Exact Test *P*-values, highlighting transcription factors whose predicted targets align most closely with the query set. Integrated results, which take into account results from all libraries, are sorted in ascending order by their scores.

### Quantitative real-time PCR assay

Total RNA of 1 µg was reverse-transcribed with HiScript III RT Super Mix for qPCR kit (R323, Vazyme, Nanjing, Jiangsu, China). The cDNA was used for real-time PCR, in which Pro Universal SYBR qPCR Master Mix and specific primers (Table S2) were utilized in a standard 2-step reaction on a 7500c Real-time PCR-detection system (Applied Biosystems, Carlsbad, CA, USA). An initial denaturation was performed at 95 °C for 30 s, followed by 40 cycles of denaturation at 95 °C for 10 s and annealing/extension at 60°C for 30 s. Finally, a melting curve analysis was conducted with the following steps: 95 °C for 15 s, 60 °C for 60 s, and 95 °C for 15 s. *ACTB* was used as an internal control gene to normalize gene expression due to its relatively stable expression in various cell types under hypoxic or normoxic conditions [31–35]. To validate the stability of *ACTB* expression under current experimental conditions, a one-way analysis of variance (ANOVA) was conducted on its cycle threshold (CT) values, showing no significant differences among the groups (Fig. S2). The 2^−∆∆CT^ method was used to calculate the relative mRNA expression of genes. 25 DEGs were enrolled in the pearson correlation analysis of quantitative real time-PCR log_2_FC and RNA seq log_2_FC.

### Intracellular ROS detection

ROS detection was performed using a DCFDA/H2DCFDA cellular ROS assay kit (ab113851, Abcam, Cambridge, UK) following the manufacturer’s instructions. Briefly, BMECs were seeded onto opaque wall perforated 96-well plates and cultured. After removing the cell culture medium, BMECs were washed using the buffer provided in the kit. 100 μL diluted DCFDA solution was added to each well, followed by incubating under the darkness for 45 min. BMECs were washed with the buffer and the fluorescence density of each well at excitation/emission = 485/535 nm was recorded by a microplate reader (Tecan, San Jose, CA, USA).

### Statistics

Cellular experiment data was visualized and statistically analyzed by Graphpad Prism (Boston, MA, USA; Version 9.0) software. Sequencing data was analyzed and visualized in R version 4.3.2 (Foundation for Statistical Computing, Vienna, Austria). All experiments were independently repeated three times, with the number of technical replicates varying by experiment as detailed in the figure captions. The data were presented as the mean ± SEM. All data were normally distributed. Comparisons between two independent groups were performed using a two-tailed unpaired *t*-test, and a one-way ANOVA with Tukey’s multiple comparison test was used to analyze differences among three or more groups. *P *< 0.05 denoted a statistically significant difference.

## Results

### Time-dependent responses of BMECs to hypoxic stress

**Fig. 1:**
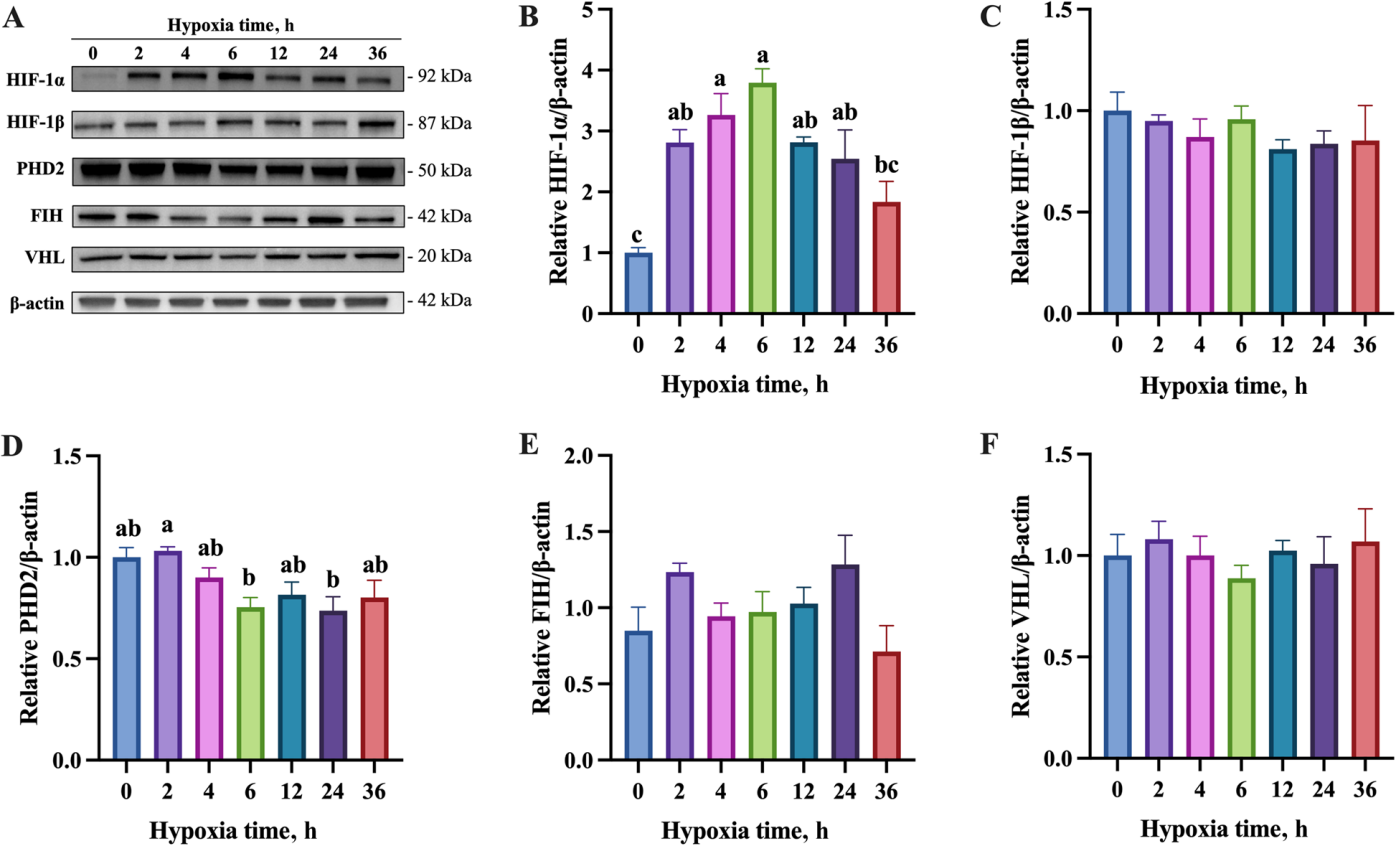
Effects of hypoxia on HIF-1 signaling in BMECs across different exposure durations. **A** Immunoblots of HIF-1α, HIF-1β, PHD2, FIH, and VHL in BMECs after exposure to hypoxia for 0, 2, 4, 6, 12, 24, and 36 h (*n* = 4). β-actin was used as the loading control. **B**–**F** Quantitative analyses of the blot density in A. Data with error bars represent mean ± SEM. Statistical significance (*P* < 0.05) marked by different lowercase letters on each bar was determined by one-way analysis of variance (ANOVA) with Tukey’s multiple comparison test

To investigate how BMECs respond to hypoxia, BMECs were exposed to hypoxia (1% O_2_, 5% CO_2_, and 94% N_2_) for various time periods. Hypoxia significantly increased the protein abundance of HIF-1α from 2 to 24 h, with a peak at 6 h (Fig. 1B). The abundance of HIF-1β (Fig. 1C) were constant across three groups, whereas Egl-9 family hypoxia inducible factor 1 (PHD2) protein abundance declined significantly at 6 h and 24 h (Fig. 1D). However, hypoxia-inducible factor-1α inhibitor (FIH) (Fig. 1E), and VHL (Fig. 1F) were not affected by hypoxia. Time points 0 h, 6 h, and 24 h were selected for subsequent experiments based on the time-series analysis, which showed these as key phases representing baseline, peak response, and sustained adaptation under hypoxia.

### Effects of hypoxia on milk synthesis and key lactation pathway proteins in BMECs

**Fig. 2:**
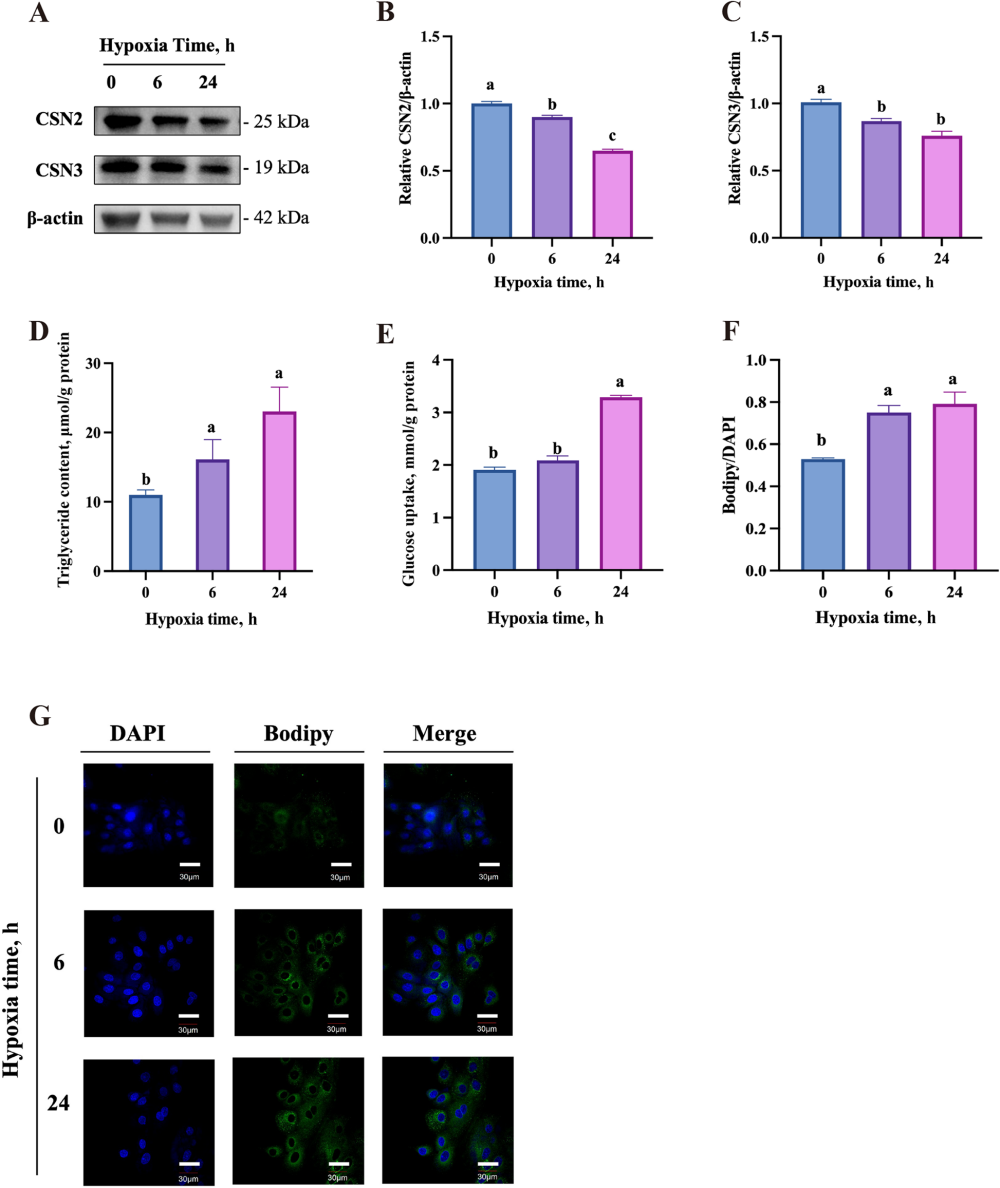
Effects of hypoxia on milk synthesis in BMECs. **A** Immunoblots of β-casein (CSN2) and κ-casein (CSN3) in BMECs after exposure to hypoxia for 0, 6, or 24 h (*n* = 3). β-actin was used as the loading control. **B** and **C** Quantitative analyses of the blot density in **A**. **D** and **E** The triglyceride content (*n* = 3) (**D**) and glucose uptake (*n* = 3) (**E**) in BMECs after exposure to hypoxia for 0, 6, or 24 h. **F** Quantitative analysis of the fluorescence stained in G. **G** The fluorescence staining of lipid droplets in BMECs after exposure to hypoxia for 0, 6, or 24 h. Blue: DAPI; Green: Bodipy. Data with error bars represent mean ± SEM. Statistical significance (*P* < 0.05) marked by different lowercase letters in individual bars was determined by ANOVA with Tukey’s multiple comparison test

**Fig. 3:**
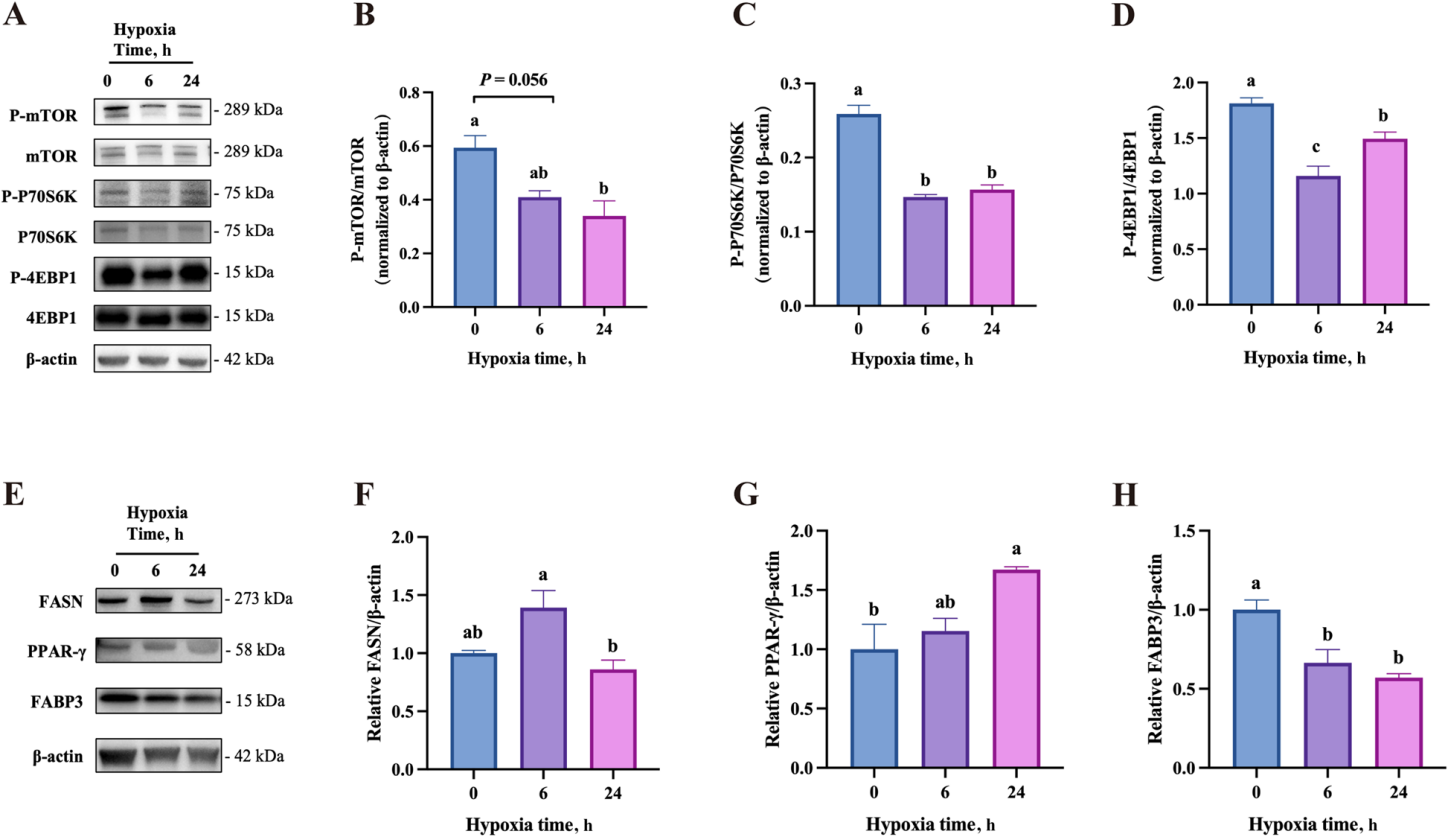
Effects of hypoxia on protein expressions of milk fat and protein synthesis regulators in BMECs. **A** Immunoblots of the phosphorylated (P) forms and total levels of the mTOR signaling proteins in BMECs after exposure to hypoxia for 0, 6, or 24 h (*n* = 3). **B**–**D** Quantitative analyses of the blot density of the phosphorylated protein abundance relative to total protein abundance in **A**. **E** Immunoblots of FASN, PPAR-γ, and FABP3 in BMECs after exposure to hypoxia for 0, 6, or 24 h (*n* = 3). **F**–**H** Quantitative analyses of the blot density in **E**. For immunoblot, β-actin was used as the loading control. Data with error bars represent mean ± SEM. Statistical significance (*P* < 0.05) marked by different lowercase letters on each bar was determined by ANOVA with Tukey’s multiple comparison test

Hypoxia significantly lowered the protein abundance of β-casein and κ-casein but raised the triglyceride content in BMECs in a time-dependent manner (Fig. 2A–D). The effect of hypoxia on glucose uptake did not depict a time-dependent manner, only 24 h hypoxia group showed significantly enhanced glucose uptake in BMECs (Fig. 2E). Lipid droplet staining by bodipy showed that both the 6 h and 24 h hypoxia groups had more lipid droplets than the normoxia group (Fig. 2F and G). In addition, cell signaling molecules related to lactation were also studied. Hypoxia lowered the phosphorylation ratio of mammalian target of rapamycin (mTOR)/ribosomal protein S6 kinase β1 (P70S6K)/eukaryotic translation initiation factor 4E binding protein 1 (4EBP1) significantly (Fig. 3A–D). Furthermore, the abundance of fatty acid synthase (FASN) and peroxisome proliferator activated receptor-γ (PPAR-γ) proteins was significantly elevated under hypoxia, but the abundance of fatty acid binding protein 3 (FABP3) was reduced (Fig. 3E–H).

### Effects of hypoxia on ATP levels and mitochondrial function in BMECs

**Fig. 4:**
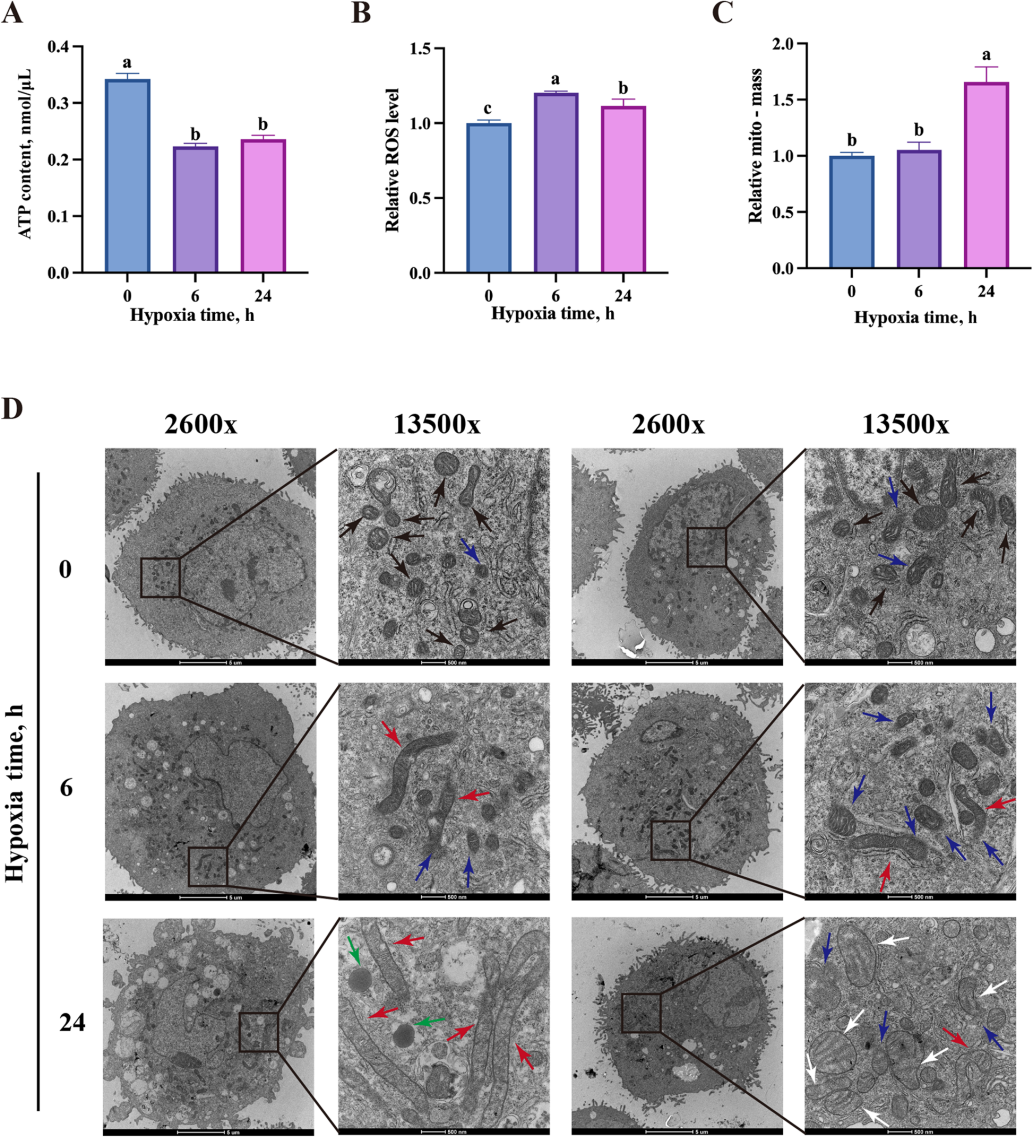
Effects of hypoxia on mitochondria structure and function in BMECs. **A** The ATP production data (*n* = 7). **B** ROS levels data (*n* = 3). **C** Mitochondrial mass data (*n* = 3). **D** Images of BMECs captured by transmission electron microscope after exposure to hypoxia for 0, 6, or 24 h. 2,600×: scale bar = 5 μm; 13,500×: scale bar = 500 nm. Black arrows: normal mitochondria; red arrows: elongated mitochondria; blue arrows: mitochondria with fuzzy margins; white arrows: swollen or crista-ruptured mitochondria; green arrows: lipid droplets. Data with error bars represent mean ± SEM. Statistical significance (*P* < 0.05) marked by different lowercase letters on individual bars was determined by ANOVA with Tukey’s multiple comparison test

Lactation places a substantial energy demand on lactating cells. To assess the impact of hypoxia, ATP levels in BMECs were measured under varying hypoxic durations. Both hypoxia treatments significantly reduced the ATP levels in BMECs (Fig. 4A). As the central site for energy production in the cell, mitochondrial structure and function were further analyzed. Elevated ROS levels and total mitochondrial mass, the indicators of mitochondrial dysfunction, were observed in BMECs under hypoxia (Fig. 4B and C). Morphological changes, including elongation, swelling, and loss of structural clarity, were also observed in mitochondria under hypoxic conditions (Fig. 4D). These findings suggest that hypoxia disrupts energy metabolism by impairing mitochondrial structure and function.

### Overview of transcriptomic findings and GO enrichment analysis

To understand the transcriptomic mechanism underlying the effects of hypoxia on lactation, we conducted transcriptomic sequencing of BMECs following 0 h, 6 h, and 24 h hypoxic incubation. The correlation between RNA-seq and qPCR for selected genes demonstrates strong concordance, with Pearson correlation coefficients of 0.759 for HP6 vs. HP0 and 0.81 for HP24 vs. HP0 (Fig. S2B). Our data showed that 0 h hypoxia (normoxia), 6 h hypoxia, and 24 h hypoxia groups were separated clearly in the principal component analysis plot (Fig. S3A). In total, 18,684 genes were detected in all groups. Compared to normoxia group, there were 371 and 939 DEGs in 6 h and 24 h hypoxia groups, respectively (Fig. S3B). We further conducted GO functional enrichment on DEGs (Fig. S4). The results showed that 379 GO terms were significantly enriched in both hypoxia groups. Specifically, both hypoxia groups significantly enriched GO terms in the oxygen level response processes including oxygen carrier activity, oxygen transport, heme binding, hypoxia response process, and oxidation–reduction process, as well as milk synthesis and metabolism including glucose metabolism and lipid metabolism (Fig. S4A and S4B). In addition, 24 h hypoxia enriched ribosome large subunit biosynthesis, which is linked to protein synthesis (Fig. S4B). Notably, compared to 6 h hypoxia, 24 h hypoxia showed more DEGs for all the above terms. Not all hypothesized genes were DEGs; the transcriptomic alterations of the hypothesized genes are described below.

### Transcriptomic profiles highlighting lactation-related key genes in BMECs under hypoxia

**Fig. 5:**
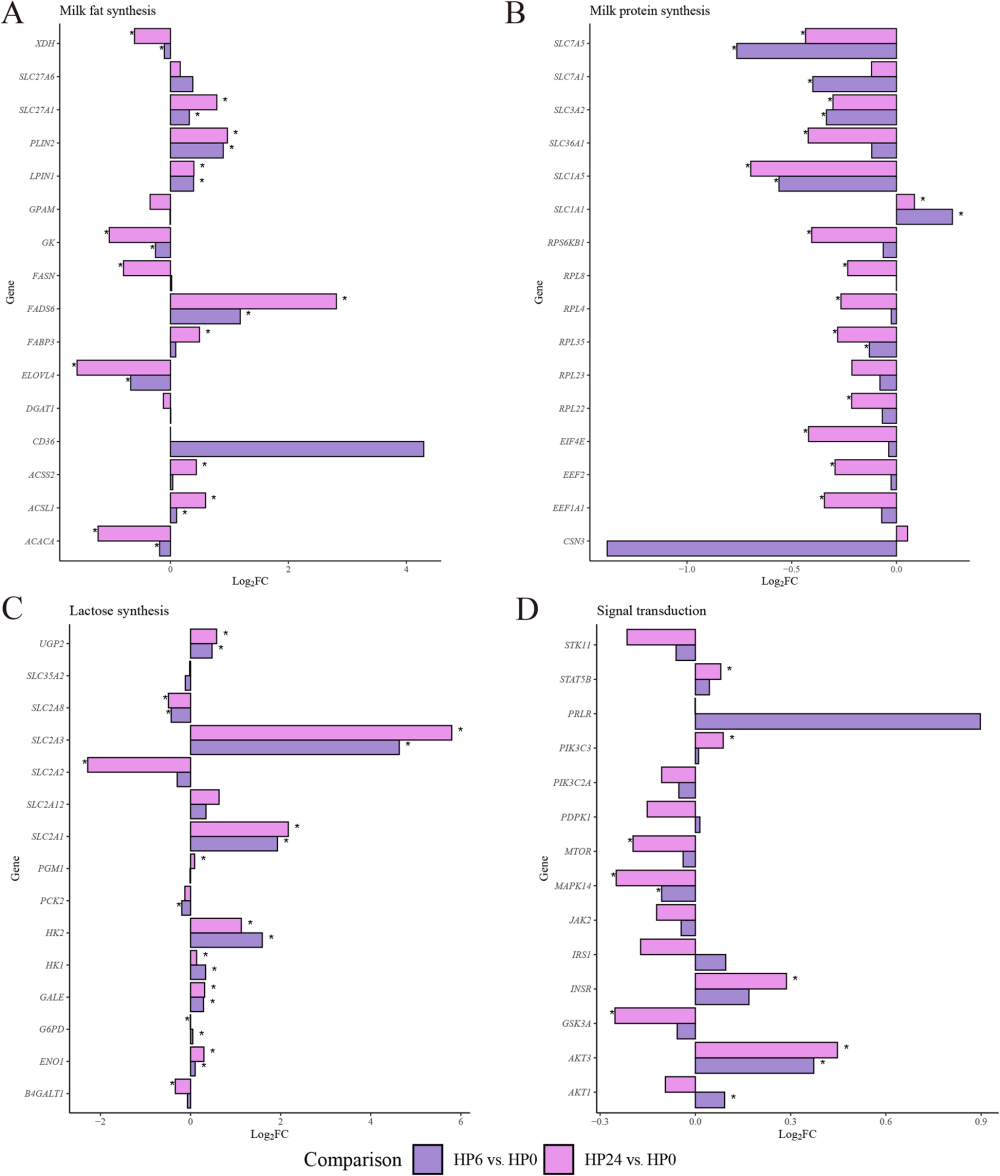
Hypoxia-induced transcriptomic changes in genes associated with lactation. **A** Milk fat synthesis related genes. The genes were classified into de novo synthesis of FA (*ACSS2*, Acyl-CoA synthetase short-chain family member 2; *FASN,* Fatty acid synthase; *FADS6*, Fatty acid desaturase 6; *GK*, Glucokinase), FA uptake from blood (*ACACA*, Acetyl-CoA carboxylase alpha; *CD36*, Cluster of differentiation 36; *SLC27A6*, Solute carrier family 27 member 6; *SLC27A1*, Solute carrier family 27 member 1; *ACSL1*, Acyl-CoA synthetase long-chain family member 1), FA transport and desaturation (*FABP3*, Fatty acid binding protein 3), TAG synthesis (*DGAT1*, Diacylglycerol O-acyltransferase 1; *LPIN1*, Lipin 1; *GPAM*, Glycerol-3-phosphate acyltransferase mitochondrial), Lipid droplet secretion (*PLIN2*, Perilipin 2; *XDH*, Xanthine dehydrogenase; *ADFP*, Adipose differentiation-related protein), and FA elongation (*ELOVL4*, Elongation of very long chain fatty acids protein 4). **B** Milk protein synthesis related genes. The genes were classified into Casein genes (*CSN3*, Casein kappa), AA Transporter (*SLC7A5*, Solute carrier family 7 member 5; *SLC7A1*, Solute carrier family 7 member 1; *SLC1A1*, Solute carrier family 1 member 1; *SLC1A5*, Solute carrier family 1 member 5; *SLC3A2*, Solute carrier family 3 member 2; *SLC36A1*, Solute carrier family 36 member 1), Translation elongation (*EEE1A1*, Eukaryotic translation elongation factor 1 alpha 1; *EEF2*, Eukaryotic translation elongation factor 2), Ribosomal function (*PRL8*, Ribosomal protein L8; *PRL4*, Ribosomal protein L4; *PRL22*, Ribosomal protein L22; *PRL23*, Ribosomal protein L23; *PRL35*, Ribosomal protein L35; *PR36KB1*, Ribosomal protein S6 kinase B1), Translation initiation (*EIF4E*, Eukaryotic translation initiation factor 4E; *EIF4EBP1*, Eukaryotic translation initiation factor 4E binding protein 1). **C** Lactose synthesis related genes. The genes were classified into Glucose uptake (*SLC2A1*, Solute carrier family 2 member 1; *SLC2A2*, Solute carrier family 2 member 2; *SLC2A3*, Solute carrier family 2 member 2; *SLC2A8*, Solute carrier family 2 member 8; *SLC2A12*, Solute carrier family 2 member 12), Lactose synthase activity (*B4GLAT1*, Beta-1,4-galactosyltransferase 1), UDP-galactose transport (*SLC35A2*, Solute carrier family 35 member A2; *UGP2*, UDP-glucose pyrophosphorylase 2; *PGM1*, Phosphoglucomutase 1), Lactose precursor production (*HK1*, Hexokinase 1; *HK2*, Hexokinase 1; *GALE*, UDP-galactose-4-epimerase; *PCK*2, Phosphoenolpyruvate carboxykinase 2; *ENO1*, Alpha-enolase; *G6PD*, Glucose-6-phosphate dehydrogenase). **D** Signal transduction related genes. *JAK2*, Janus kinase 2; *STAT5B*, Signal transducer and activator of transcription; *PRLR*, Prolactin receptor; *AKT1*, AKT serine/threonine kinase 1; *AKT3*, AKT serine/threonine kinase 3; *MTOR*, Mechanistic target of rapamycin; *GSK3A*, Glycogen synthase kinase 3 alpha; *INSR*, Insulin receptor; *IRS1*, Insulin receptor substrate 1; *MAPK14*, Mitogen-activated protein kinase 14; *PDPK1*, Phosphoinositide-dependent protein kinase 1; *PIK3C2A*, Phosphoinositide-3-kinase catalytic subunit type 2 alpha; *STK11*, Serine/threonine kinase 11

Transcriptomic analysis revealed changes in genes involved in key processes of milk fat synthesis, including de novo fatty acid synthesis, fatty acid uptake, transport, and desaturation, triacylglycerol synthesis, and lipid droplet formation (Fig. 5A). Genes such as *SLC27A1*, *PLIN2*, *LPIN1*, *FADS6*, and *ACSL1* were upregulated in both HP6 vs. HP0 and HP24 vs. HP0 comparisons, while *XDH*, *ELOVL4*, *GK* and *ACACA* were consistently downregulated. Time-specific regulation was observed, with *FABP3* and *ACSS2* upregulated only in HP24 vs. HP0, and *FASN* downregulated exclusively in HP24 vs. HP0. Genes associated with milk protein synthesis were grouped by casein synthesis, amino acid transport, and translation processes, including elongation, initiation, and ribosomal function (Fig. 5B). Among these, *SLC7A5, SLC3A2, SLC1A5,* and *RPL35* were consistently downregulated in both HP6 vs. HP0 and HP24 vs. HP0 comparisons, while *SLC1A1* was the only gene upregulated in both comparisons. Time-specific changes included *SLC7A1*, which was downregulated exclusively in HP6 vs. HP0, and *RPS6KB1*, *RPL8*, *RPL4*, *RPL22*, *EIF4E*, *EEF2*, and *EEF1A1*, which were downregulated only in HP24 vs. HP0. Transcriptomic changes were observed in genes related to glucose uptake, lactose synthase activity, and the production and transport of lactose precursors (Fig. 5C). Genes such as *UGP2*, *SLC2A3*, *SLC2A1*, *HK2*, *HK1*, *GALE*, and *ENO1* were upregulated in both HP6 vs. HP0 and HP24 vs. HP0 comparisons. In contrast, *SLC2A2* and *B4GALT1* were downregulated exclusively in HP24 vs. HP0, while *PCK2* was downregulated only in HP6 vs. HP0.

The RNA abundance of key genes associated with lactation signal transduction was analyzed by sequencing (Fig. 5D). Among these, *MAPK14* was the only gene consistently downregulated in both comparisons, while *AKT3* was upregulated in both comparisons. In HP24 vs. HP0, *MTOR* and *GSK3A* were downregulated, while *INSR, STAT5B,* and *PIK3C3* were upregulated. Additionally, *AKT1* was exclusively upregulated in HP6 vs. HP0.

### Transcriptomic profiles highlighting energy metabolism key genes in BMECs under hypoxia

**Fig. 6:**
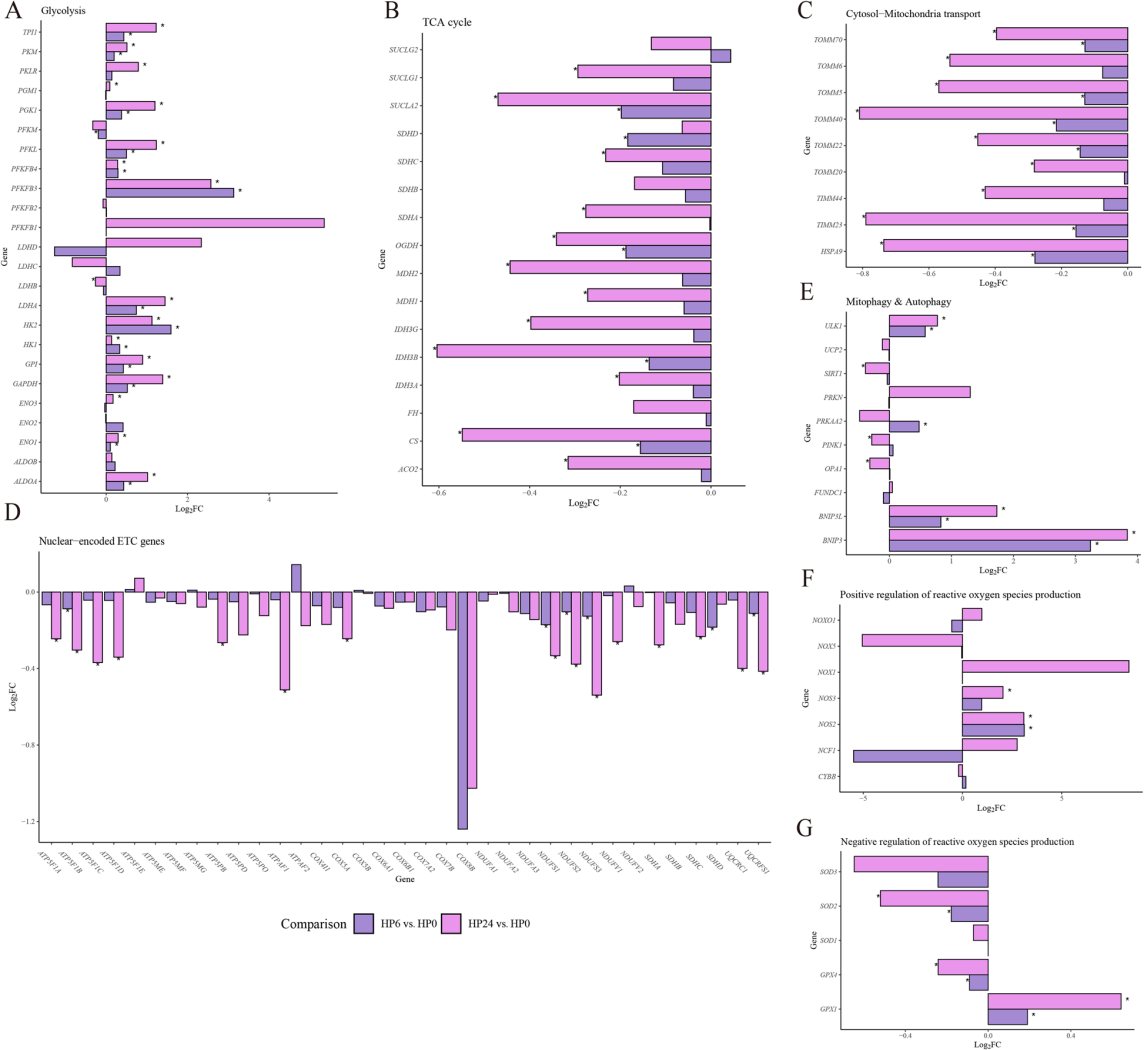
Hypoxia-induced transcriptomic changes in genes associated with energy metabolism. **A** Glycolysis. *HK1*, Hexokinase 1; *HK2*, Hexokinase 2; *GP1*, Glycogen phosphorylase 1; *PFKB1*, 6-phosphofructokinase 1; *PFKB2*, 6-Phosphofructokinase 2; *PFKB3*, 6-Phosphofructokinase 3; *PFKB4*, 6-Phosphofructokinase 4; *PFKL*, Phosphofructokinase L; *PFKM*, Phosphofructokinase M; *ALDOB*, Aldolase B; *ALDOA*, Aldolase B; *TPI1*, Triosephosphate isomerase 1; *GAPDH*, Glyceraldehyde-3-phosphate dehydrogenase; *PGK1*, Phosphoglycerate kinase 1; *PGM1*, Phosphoglucomutase 1; *ENO1*, Alpha-enolase; *ENO2*, Beta-enolase; *ENO3*, Gamma-enolase; *PKM*, Pyruvate kinase M1/M2; *PKLR*, Pyruvate kinase L/R; *LDHA*, Lactate dehydrogenase A; *LDHB*, Lactate dehydrogenase B; *LDHC*, Lactate dehydrogenase C; *LDHD*, Lactate dehydrogenase D. **B** TCA cycle. *CS*, Citrate synthase; *ACO2*, Aconitase 2; *IDH3A*, Isocitrate dehydrogenase 3 alpha; *IDH3G*, Isocitrate dehydrogenase 3 gamma; *IDH3B*, Isocitrate dehydrogenase 3 beta; *OGDH*, Oxoglutarate dehydrogenase; *SUCLG2*, Succinate-CoA ligase GDP-forming beta subunit; *SUCLA2*, Succinate-CoA ligase alpha subunit; *SUCLG1*, Succinate-CoA ligase beta subunit; *SDHA*, Succinate dehydrogenase complex flavoprotein subunit A; *SDHB*, Succinate dehydrogenase complex iron-sulfur subunit B; *SDHC*, Succinate dehydrogenase complex subunit C; *SDHD*, Succinate dehydrogenase complex subunit D; *FH*, Fumarate hydratase; *MDH2*, Malate dehydrogenase 2; *MDH1,* Malate dehydrogenase 2. **C** Cytosol-Mitochondria transport The genes were classified into TOM complex (*TOMM20*, Translocase of outer mitochondrial membrane 20; *TOMM40*, Translocase of outer mitochondrial membrane 44; *TOMM22*, Translocase of outer mitochondrial membrane 22; *TOMM70*, Translocase of outer mitochondrial membrane 70; *TOMM5*, Translocase of outer mitochondrial membrane 5; *TOMM6*, Translocase of outer mitochondrial membrane 6), TIM complex (*TIMM23*, Translocase of inner mitochondrial membrane 23; *TIMM44*, Translocase of inner mitochondrial membrane 44), HSP (*HSPA9*, Heat shock 70 kDa protein 9). **D** Nuclear-encoded ETC genes. The genes were classified into Complex I (*NDUFV1*, NADH dehydrogenase [ubiquinone] 1 alpha subcomplex, 1; *NDUFV2*, NADH dehydrogenase [ubiquinone] 1 alpha subcomplex, 2; *NDUFS1*, NADH dehydrogenase [ubiquinone] 1 beta subcomplex, 1; *NDUFS2*, NADH dehydrogenase [ubiquinone] 1 beta subcomplex, 2; *NDUFS3,* NADH dehydrogenase [ubiquinone] 1 beta subcomplex, 3; *NDUFA1*, NADH dehydrogenase [ubiquinone] 1 alpha subcomplex, 1; *NDUFA2*, NADH dehydrogenase [ubiquinone] 1 alpha subcomplex, 2; *NDUFA3*, NADH dehydrogenase [ubiquinone] 1 alpha subcomplex, 3), Complex II (*SDHA*, Succinate dehydrogenase complex flavoprotein subunit A; *SDHB*, Succinate dehydrogenase complex iron-sulfur subunit B; *SDHC*, Succinate dehydrogenase complex subunit C; *SDHD*, Succinate dehydrogenase complex subunit D), Complex III (*UQCRC1*, Ubiquinol-cytochrome c reductase complex core protein 1; *UQCRFS1*, Ubiquinol-cytochrome c reductase complex supernumerary subunit 1), Complex IV (*COX4I1*, Cytochrome c oxidase subunit 4 isoform 1; *COX5A*, Cytochrome c oxidase subunit 5A; *COX5B*, Cytochrome c oxidase subunit 5B; *COX6A1*, Cytochrome c oxidase subunit 6A1; *COX6B1*, Cytochrome c oxidase subunit 6B1; *COX7A2*, Cytochrome c oxidase subunit 7A2; *COX7B*, Cytochrome c oxidase subunit 7B; *COX8B*, Cytochrome c oxidase subunit 8B), ATP synthase (*ATP5F1A*, ATP synthase F1 subunit alpha; *ATP5F1B*, ATP synthase F1 subunit beta; *ATP5F1C*, ATP synthase F1 subunit gamma; *ATP5F1D*, ATP synthase F1 subunit delta; *ATP5F1E*, ATP synthase F1 subunit epsilon; *ATP5ME*, ATP synthase mitochondrial E; *ATP5MF*, ATP synthase mitochondrial; *ATP5MG*, ATP synthase mitochondrial; *ATP5PB*, ATP synthase F0 subunit b; *ATP5PD*, ATP synthase F0 subunit d; *ATP5PO*, ATP synthase F0 subunit o; *ATPAF1*, ATP synthase assembly factor 1; *ATPAF2*, ATP synthase assembly factor 2). **E** Mitophagy & Autophagy. *PINK1*, PTEN-induced kinase 1; *PRKN*, Parkin RBR E3 ubiquitin protein ligase; *FUNDC1*, FUN14 domain containing 1; *BNIP3*, BCL2/adenovirus E1B 19kDa interacting protein 3; *BNIP3L*, BCL2/adenovirus E1B 19kDa interacting protein 3-like; *UCP2*, Uncoupling protein 2; *PRKAA2*, Protein kinase AMP-activated catalytic subunit alpha 2; *SIRT1*, Sirtuin 1; *ULK1*, Unc-51 like autophagy activating kinase 1; *OPA1*, Mitochondrial dynamin-like GTPase. **F** Positive regulation of reactive oxygen species production. *NOX1*, NADPH oxidase 1; *CYBB*, Cytochrome b-245 beta chain; *NCF1*, Neutrophil cytosolic factor 1; *NOXO1*, NADPH oxidase organizer 1; *NOX5*, NADPH oxidase 5; *NOS2*, Nitric oxide synthase 2; *NOS3*, Nitric oxide synthase 3. **G** Negative regulation of reactive oxygen species production. *SOD1*, Superoxide dismutase 1; *SOD2*, Superoxide dismutase 2; *SOD3*, Superoxide dismutase 3; *GPX1*, Glutathione peroxidase 1; *GPX4*, Glutathione peroxidase 4

The transcriptomic abundance of genes related to glycolysis, TCA cycle, cytosol-mitochondria transport and nuclear-encoded electronic transport chain (ETC) complex genes was shown in Fig. 6. The genes encoding for glycolysis enzymes were mostly upregulated in both comparisons (Fig. 6A). In particular, *TPI1*, *PKM*, *PGK1*, *PFKL*, *PFKFB4*, *HK1*, *GPI*, *GAPDH*, *ENO1*, *ALDOA*, *HK2*, *LDHA* and *PFKFB3* were upregulated significantly in both comparison (Fig. 6A). *ENO3* was upregulated while *LDHB* was downregulated only in HP24 vs. HP0 and *PFKM* was downregulated only in HP6 vs. HP0 (Fig. 6A). Most hypothesized TCA cycle enzyme encoding genes were downregulated in both comparison (Fig. 6B). Specifically, *SUCLA2*, *OGDH*, *IDH3B* and *CS* were significantly downregulated in both comparison while *SUCLG1*, *SDHA*, *SDHC*, *MDH1*, *IDH3G*, *IDH3A* and *ACO2* were downregulated exclusively in HP24 vs. HP0 (Fig. 6B). Additionally, *SDHD* was downregulated only in HP6 vs. HP0 (Fig. 6B). For genes associated with cytosol-mitochondrial transport, all hypothesized genes were downregulated in both comparisons (Fig. 6C). In the HP24 vs. HP0, genes encoding the TOM complex, TIM complex, and HSP70 protein exhibited greater fold changes compared to their corresponding genes in the HP6 vs. HP0 (Fig. 6C).

For nuclear-encoded genes associated with electronic transport chain, most genes related to the ETC complex were downregulated, with 24 h of hypoxia resulting in a greater number of significantly affected genes (Fig. 6D). NADH dehydrogenase related genes such as *NDUFS3, NDUFS2* and *NDUFS1* were consistently downregulated in both comparisons. *NDUFV1* was only downregulated after 24 h hypoxic treatment (Fig. 6D). Both *UQCRFS1* and *UQCRC1* related to ubiquinol-cytochrome c reductase were downregulated in HP24 vs. HP0 (Fig. 6D). Except *COX5A* was downregulated significantly in HP24 vs. HP0 among the cytochrome c oxidase related genes, the rest cytochrome c oxidase related genes did not show significant alteration (Fig. 6D). ATP synthase related genes such as *ATPAF1*, *ATP5PB*, *ATP5F1D*, *ATP5F1C* and *ATP5F1A* were exclusively downregulated in HP24 vs. HP0 while *ATP5F1B* was downregulated in both comparisons (Fig. 6D). Given the observed mitochondrial structural impairment and ROS production in previous experiments, we also investigated the transcriptomic changes in genes associated with mitophagy, autophagy and ROS production (Fig. 6E–G). As a bordar regulator for autophagy, *ULK1* was significantly upregulated in both comparisons (Fig. 6E). As essential mediators of hypoxia-induced and ubiquitin-independent mitophagy, *BNIP3* and *BNIP3L* were significantly upregulated in both comparisons (Fig. 6E). For positive regulation of ROS production, *NOS2* were downregulated in either HP6 vs. HP0 or HP24 vs. HP0 comparisons. For the negative regulation of ROS production, *SOD2* and *GPX4* exhibited downregulation but *GPX1* was upregulated in both comparisons (Fig. 6F).

### HIF-1α is involved for hypoxia effects of TAG synthesis and glucose uptake in BMECs

**Fig. 7:**
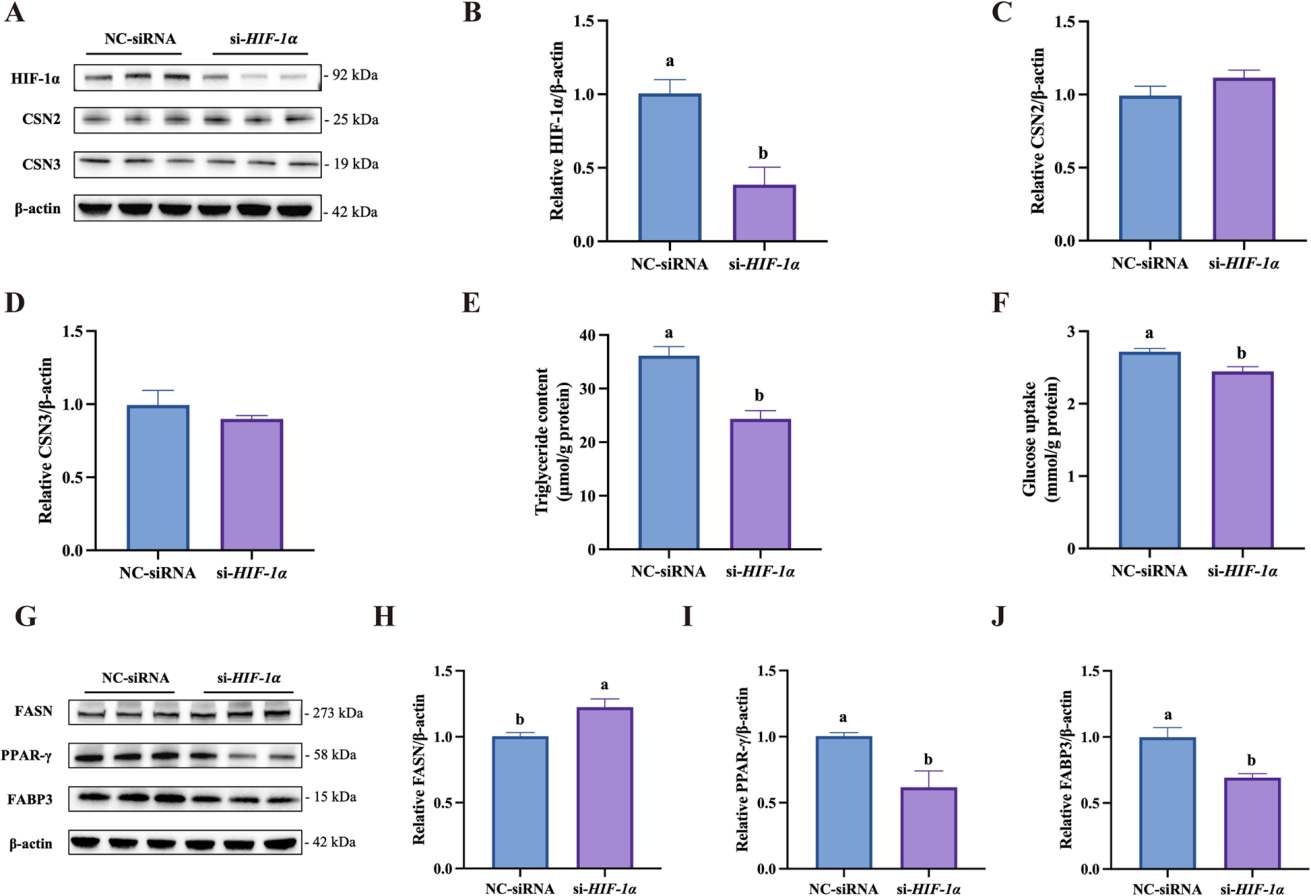
Effects of *HIF-1α *knockdown on milk synthesis in BMECs. **A** Immunoblots of HIF-1α , β-casein (CSN2), and κ-casein (CSN3) in BMECs under 24 h hypoxia and transfected with either the control (NC) siRNA or *HIF-1*α siRNA (*n* = 3). **B**–**D** Quantitative analyses of the blot densities in **A**. **E** and **F** The triglyceride content (*n* = 3) (**E**) and glucose uptake (*n* = 3) (**F**) in BMECs under 24 h hypoxia and transfected with either NC siRNA or *HIF-1α *siRNA. **G** Immunoblots of FASN, PPAR-γ, and FABP3 in BMECs under 24 h hypoxia and transfected with either NC siRNA or *HIF-1α *siRNA (*n* = 3). **H**–**J** Quantitative analyses of the blot densities in G. For immunoblots, β-actin was used as the loading control. Data with error bars represent mean ± SEM. Statistical significance (*P* < 0.05) marked by different lowercase letters on individual bars was determined by two-tailed unpaired *t*-test

As 24 h hypoxic treatment exhibited a greater number of DEGs, we did an exploratory analysis to enrich transcription factors using the DEGs in HP24 vs. HP0. 1632 transcription factors were identified and the top 30 transcription factor enriched were shown in Fig. S5. As the most importance marker protein under hypoxia, HIF-1α ranked at 28 with 45 target genes overlapped with the library. To investigate if HIF-1α is involved in milk synthesis in BMECs, we carried out *HIF-1*α knockdown in BMECs using *HIF-1*α siRNA. *HIF-1*α knockdown had no effect either on the protein abundance of β-casein and κ-casein (Fig. 7A–D) or the phosphorylation ratio of mTOR/P70S6K/4EBP1 proteins (Fig. S6). However, *HIF-1*α knockdown significantly decreased triglyceride content (Fig. 7E) and glucose uptake (Fig. 7F) in BMECs. Meanwhile, *HIF-1*α knockdown significantly decreased the protein abundance of PPAR-γ and FABP3 while increasing FASN expression (Fig. 7G–J).

## Discussion

**Fig. 8:**
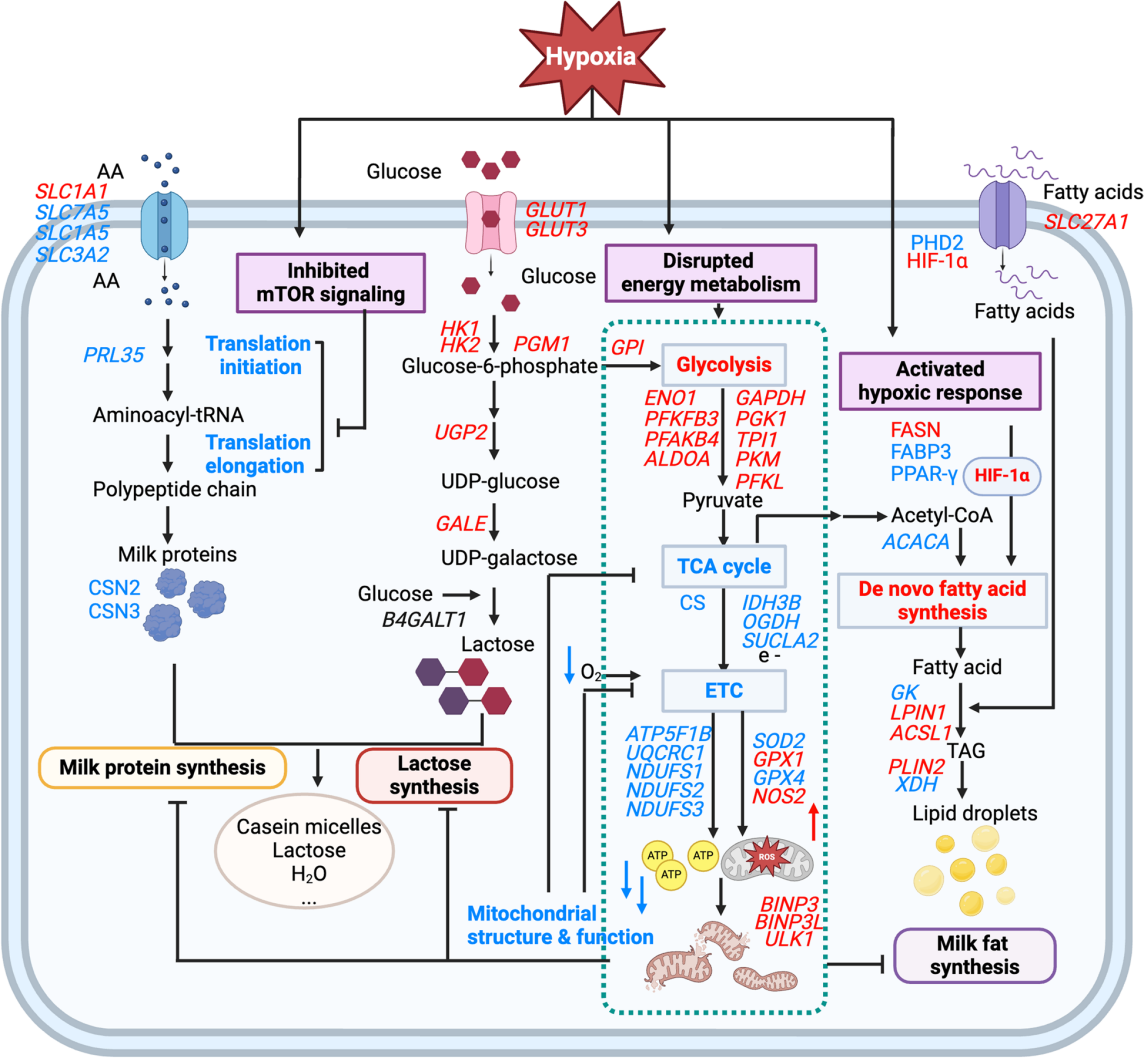
Scheme of the mechanisms of hypoxia’s effects on milk synthesis in BMECs. The red color represents up-regulated genes, proteins, and processes; and the blue color represents down-upregulated genes, proteins, and processes. The italic font represents genes, and the normal font represents proteins and processes

In this study, we investigated the effects of hypoxia on lactation capacity using an in vitro BMEC model, emphasizing the differential transcriptomic expression of genes associated with lactation and energy metabolism, as well as the pivotal role of HIF-1α in regulating key proteins involved in lactation pathways (Fig. 8). The venous blood oxygen concentration of dairy cows is 11% [1]. In this study, 1% oxygen concentration has be employed to mimic the extreme conditions that may impair lactation [1]. A time series experiment was conducted to identify the appropriate length of hypoxic treatment. 0 h, 6 h and 24 h were selected because the representative alterations of HIF-1α protein abundance in these time points. Our findings suggested that PHD2 may play a more significant role in sensing oxygen than FIH and VHL in BMECs. The quantity of FIH and VHL proteins did not vary between different durations of hypoxic exposure, while PHD2 expression changed significantly. This might be attributed to the oxygen affinity differences. PHD2 is more sensitive to oxygen levels because it has a lower affinity to oxygen with a *K*_*m*_ of roughly 230–250 μmol/L , whereas FIH has a high oxygen affinity with a *K*_*m*_ of 90 μmol/L [36, 37].

Our study highlighted that hypoxia disrupted milk protein synthesis in BMECs. β-Casein and κ-casein are essential to maintain an appropriate protein composition and yield in dairy products [38, 39]. We found that hypoxia decreased β-casein and κ-casein protein abundance in BMECs. This might attribute to the altered phosphorylation ratio of mTOR/P70S6K/4EBP1. The mTOR signaling suppression could inhibit eukaryotic translation initiation factor 4E assembly which could lead to decreased mRNA translation and subsequent protein production [40, 41]. Studies showed that hypoxia can promote the expression of eukaryotic elongation factor 2 kinase (eEF2K) which is the downstream of mTOR signaling function as an inactivator of eEF2 and finally inhibits translation elongation [42, 43]. We also identified significant transcriptomic downregulation of translation initiation factor coding gene *EIF4E* and translation elongation factor encoding gene *EEF2* and *EEF1A1* after 24 h hypoxic incubation. However, in our study, *HIF-1α* knockdown didn’t influence the effects of hypoxia on casein expression and mTOR signaling activation suggesting that mTOR signaling may interact with other regulators, rather than HIF-1α to modulate casein expression under hypoxic conditions. Additionally, we identified significant downregulation of multiple ribosomal protein coding genes like *RPL8, RPL4, RPL35, RPL22, RPS6KB1* after 24 h hypoxic treatment, suggesting a possibly disrupted ribosomal function under hypoxia. Besides, transcriptomic abundance of several amino acid transporters has been decreased after both 6 h and 24 h hypoxic exposure, indicating the availability of amino acid may decrease under hypoxia.

The majority of milk fat is triglycerides that are synthesized by mammary epithelial cells [44]. In our study, hypoxia increased triglyceride content and the number of lipid droplets in a time-dependent way. Our transcriptomic analysis revealed an upregulation of *FADS6*, a gene involved in polyunsaturated fatty acid synthesis in milk [45], in the hypoxia groups, suggesting that hypoxia may enhance polyunsaturated fatty acid production. Genes involved in fatty acid de novo synthesis such as *GK* and *ELOVL4* were significantly downregulated in both comparison. *FASN* is a key gene involved in milk fat synthesis. In our transcriptomic analysis, *FASN* expression did not significantly change in HP6 vs. HP0 but was significantly decreased in HP24 vs. HP0. Our molecular experiments also showed inconsistent FASN protein expression between HP6 and HP24, where FASN protein abundance significantly increased in HP6 group but did not change in HP24 group. A previous study on human mesenchymal stem cells reported time-dependent expression of FASN protein under hypoxia [46]. Interestingly, FASN expression appears to correspond to the duration of hypoxic exposure, with the highest expression observed when the strongest hypoxic response was induced [46]*.*

Previous study found increase lipid storage in cells under hypoxia may derive from accelerated fatty acid uptake instead of fatty acid de novo synthesis [47]. Similarly, in this study, genes involved in de novo fatty acid synthesis like *GK* and *ELOVL4* were downregulated significantly but genes involved in fatty acid uptake related genes like *FABP3*, *ASCL1*, *SLC27A6* and *SLC27A1* were upregulated significantly. In addition, enhanced triglycerides synthesis in BMECs under hypoxia could be associated with PPAR-γ, a key regulatory factor in fatty acid synthesis. Particularly, the elevated triglyceride content and expression of PPAR-γ were eliminated after *HIF-1α* knockdown, highlighting that PPAR-γ might be an essential target of the regulation of milk fat synthesis by HIF-1α.

Glucose is essential for lactose synthesis which maintains milk osmotic pressure and determines milk yield. In this study, we discovered that hypoxia enhanced glucose absorption in BMECs. This was consistent with prior research that 12 h hypoxia treatment significantly increased glucose uptake and glucose transporter protein type 1 (*GLUT1*) mRNA levels in BMECs [17]. Our study also identified increased expression of sugar transporters including *SLC2A1*, *SLC2A2* and *SLC2A3* in hypoxic cells. Consistently, the elevated glucose uptake in hypoxic BMECs was abolished after *HIF-1α* knockdown as also shown in the previous study [48]. Although multiple lactose precursor production genes were identified to be significantly upregulated in both groups, only *UGP2*, *GALE* and *PGM1* were specific to galactose synthesis while other genes such as *HK1*, *HK2* and *ENO1* were also involved in glycolysis. The lactose synthesis was carried out by a heterodimer assembled from beta-1,4-galactosyltransferase (encoded by *B4GALT1*) and α-lactalbumin [49]. Although the interconversion of glucose and lactose seems to be improved, the transcriptomic abundance of *B4GALT1* did not change significantly in HP6 vs. HP0 and even decreased in HP24 vs. HP0. This suggests that the enhanced glucose uptake may be redirected toward other functions, such as energy production. This redirection could be attributed to the oxygen deprivation caused low energy production efficiency. Under hypoxic conditions, cells mainly rely on the less efficient glycolysis pathway to support their survival and function [2].

We demonstrated this through comparing gene expression related to energy metabolism, where we found glycolysis was promoted but TCA cycle, cytosol-mitochondrial transport, oxidative phosphorylation was suppressed at transcriptomic level under hypoxia. The transcriptomic changes of key enzymes in each step in glycolysis have been upregulated at varied extent. It is important to note *PFKFB3* which has been upregulated in both comparison with a high fold change. This is consistent with previous research where *PFKFB3* has been shown to be stimulated by hypoxia in various models due to the existence of hypoxic response element [50–52] in the promoter region. *PFKBF3* encodes fructose-2,6-biphosphatase 3, which acts a key positive regulator for the rate-limiting enzyme phosphofructose kinase by promoting fructose-2,6-biphosphate synthesis [53]. Similarly, Hexokinase, which also has hypoxic response element [54–56], is another glycolysis rate-limiting enzyme that catalyzes the conversion of glucose to glucose-6-phosphate. Both *HK1* and *HK2* has been upregulated significantly in both comparisons. We also identified upregulated transcriptomic abundance of lactate dehydrogenase *LDHA*. LDHA converts pyruvate into lactate and simultaneously regenerate NAD^+^ from NADH, which is crucial for the maintenance of glycolysis under low oxygen conditions [57, 58].

The transcriptomic changes of genes involved in every step in the TCA cycle has been downregulated except the reaction converting fumarate to malate catalyzed by fumarase [59–62]. Regarding genes involved in nuclear-encoded ETC genes, multiple genes related to complex I, complex II and ATP synthase were downregulated under hypoxia, indicating an overall suppression of the oxidative phosphorylation and low efficiency of energy production at the transcription level. The inhibition of oxidative phosphorylation might be attributed to decreased efficiency of cytosol-mitochondrial transport. Most of the mitochondrial proteins, including those constituting the ETC, are encoded in the nucleus, synthesized by ribosomes in the cytosol and imported into mitochondria through TOM complex on the outer mitochondrial membrane and TIM complex on the inner mitochondrial membrane [59]. Additionally, *HSPA9* encodes for a mitochondria-located HSP70 protein, acting as a molecular chaperone, that also participates in ETC protein transportation across mitochondria membrane [60–62]. Six TOM complex genes, two TIM complex genes, and *HSPA9* were downregulated in hypoxic conditions.

Due to the lack of oxygen as the final electron recipient, hypoxia-induced reduced ETC activity could disrupt the normal flow of electrons and result in insufficient electron transfer, electron accumulation and leakage [63]. The surplus electrons can prematurely react with molecular oxygen leading to the formation of reactive oxygen species under hypoxic conditions [64–67]. In the transcriptome, *NOS3* and *NOS2* were upregulated under hypoxia. This further indicates the existence of oxidative stress in hypoxic conditions. Oxidative damage to mitochondrial membrane can result in alteration in the membrane fluidity and permeability and disruption in the mitochondrial membrane integrity. This align with our observation under transmission electronic microscopy where more mitochondria have been identified with various structure deficits. We speculate the mitochondria activated mitophagy and autophagy to clear excessive impaired mitochondria as the *ULK1, BNIP3* and *BNIP3L* were upregulated under hypoxia. However, the study did not assess autophagy or mitophagy directly. Future studies in this area are necessary to clarify if the hypoxia-induced lactation phenotype alteration is associated with mitophagy or autophagy. Aside from hypoxia-induced energy metabolism impairment, apoptosis might also contribute to lactation disruption under hypoxia as hypoxia-induced apoptosis was observed in BMECs in a previous study [1]. Additionally, milk synthesis is a complex biological process; however, this study focused only on the key aspects of the synthesis of its main components. Future studies using lactation cell or animal models will be invaluable for exploring various aspects of milk component synthesis, providing a more comprehensive understanding.

## Conclusions

This study highlights the impacts of hypoxia on milk synthesis in BMECs. Hypoxia disrupts milk protein synthesis by reducing mTOR/P70S6K/4EBP1 phosphorylation and downregulating genes associated with amino acid uptake, translation, and ribosomal function. It promotes triglyceride synthesis by enhancing fatty acid uptake and upregulating the expression of key regulatory proteins, including FASN and PPAR-γ. While hypoxia increases glucose uptake, the transcriptomic downregulation of lactose synthesis enzymes suggests a redirection of glucose toward glycolysis rather than lactose production. Hypoxia impaired energy production in BMECs through reducing the transcriptomic abundance of genes involved in the TCA cycle, cytosol-mitochondrial transport, and ETC activity. Hypoxia also induced ROS elevation, contributing to mitochondrial structural and functional impairment. Notably, while glucose uptake and triglyceride synthesis are HIF-1α-dependent, the reduction in milk protein synthesis is independent of HIF-1α. These findings offer critical insights into the molecular mechanisms of how hypoxic stress impacts lactation.

## Supplementary Information

Additional file 1: Fig. S1. Scheme of the study workflow.

Additional file 2: Fig. S2. qRT-PCR verification of DEGs from RNA sequencing. (A) Analysis of ACTB CT values across three groups (*n* = 4). Data with error bars represent mean ± SEM. (B) Pearson correlation analysis of the log2FC of DEGs tested by qRT-PCR and RNA sequencing.

Additional file 3: Fig. S3. Transcriptomic signatures of BMECs under hypoxia for 0, 6, 24 h. (A) PCA (principal component analysis) score plots. (B) Summary of the numbers of up-regulated and down-regulated differentially expressed genes (DEGs) in both hypoxia groups. (C and D) Volcano plots of genes detected in 6 h hypoxia group (HP6) (C) and 24 h hypoxia group (HP24) (D) compared to normoxia group. (E and F) Heatmap of the transcriptome in 6 h hypoxia (E) and 24 h hypoxia (F) groups compared to the normoxia group. The data underlying this figure can be found in the Table S3 and Table S4.

Additional file 4: Fig. S4. GO enrichment of DEGs in BMECs under hypoxia. (A) and (B) shows the GO enrichment results in hypoxia for 6 h or 24 h respectively, compared to normoxia. The data underlying this figure can be found in the Table S5 and Table S6.

Additional file 5: Fig. S5. Transcription factor enrichment of DEGs in HP24 vs. HP0 comparison.

Additional file 6: Fig. S6. Effects of *HIF-1α* knockdown on mTOR signaling proteins phosphorylation in BMECs. (A) Immunoblots of the phosphorylated forms (P) and total levels (T) of the mTOR signaling proteins in BMECs under 24 h hypoxia and transfected with either a control (NC) siRNA or *HIF-1α *siRNA (*n* = 3). (B–D) Quantitative analyses of the blot densities of the P/T forms of the mTOR signaling proteins in A. Data with error bars represent mean ± SEM. Statistical significance (*P* < 0.05) was determined by a two-tailed unpaired t-test.

Additional file 7: Table S1. Antibodies for immunoblot.

Additional file 8: Table S2. Primers used for quantitative real-time PCR.

Additional file 9: Table S3. The DEGs in BMECs after 6 h hypoxia exposure vs. normoxia group.

Additional file 10: Table S4. The DEGs in BMEC after 24 h hypoxia exposure vs. normoxia group.

Additional file 11: Table S5. GO enrichment results of DEGs in BMEC under 6 h hypoxia vs. normoxia exposure.

Additional file 12: Table S6. GO enrichment results of DEGs in BMEC under 24 h hypoxia vs. normoxia exposure.

## Data Availability

The data generated in the current study are available from the corresponding author on reasonable request.

## Abbreviations

4EBP1: Eukaryotic translation initiation factor 4E binding protein 1
B4GALT1: Beta-1,4-galactosyltransferase 1
BMECs: Bovine mammary epithelial cells
BNIP3: Bcl2 interacting protein 3
ChEA3: ChIP-X Enrichment Analysis
DEGs: Differential expressed genes
DMEM: Dulbecco's modified Eagle's medium
EEF2: Eukaryotic translation elongation factor 2
ETC: Electronic transport chain
ELOVL4: Elongation of very long chain fatty acids protein 4
FABP3: Fatty acid binding protein 3
FASN: Fatty acid synthase
FIH: Factor inhibiting hypoxia-inducible factor 1α
GK: Glucokinase
GO: Gene ontology
HIF-1α: Hypoxia-inducible factor 1α
HK1: Hexokinase 1
HK2: Hexokinase 2
LDHA: Lactate dehydrogenase A
mTOR: Mammalian target of rapamycin
NADH: Nicotinamide adenine dinucleotide
P70S6K: Ribosomal protein S6 kinase β1
PBS: Phosphate-buffered saline
PFKFB3: 6-Phosphofructo-2-kinase
PHD2: Egl-9 family hypoxia inducible factor 1
PPAR-γ: Peroxisome proliferator activated receptor-γ
ROS: Reactive oxygen species
TCA: Tricarboxylic acid
TOM: Translocase of the outer mitochondrial membrane
TIM: Translocase of the inner mitochondrial membrane
VHL: Von hippel-lindau tumor suppressor
XDH: Xanthine dehydrogenase
