# Supplementary figures and images for: Effects of hypoxia stress on the milk synthesis in bovine mammary epithelial cells
Source: J Anim Sci Biotechnol. 2025 Mar 7;16:37. doi: 10.1186/s40104-025-01174-0 (PMC11887346; doi:10.1186/s40104-025-01174-0)

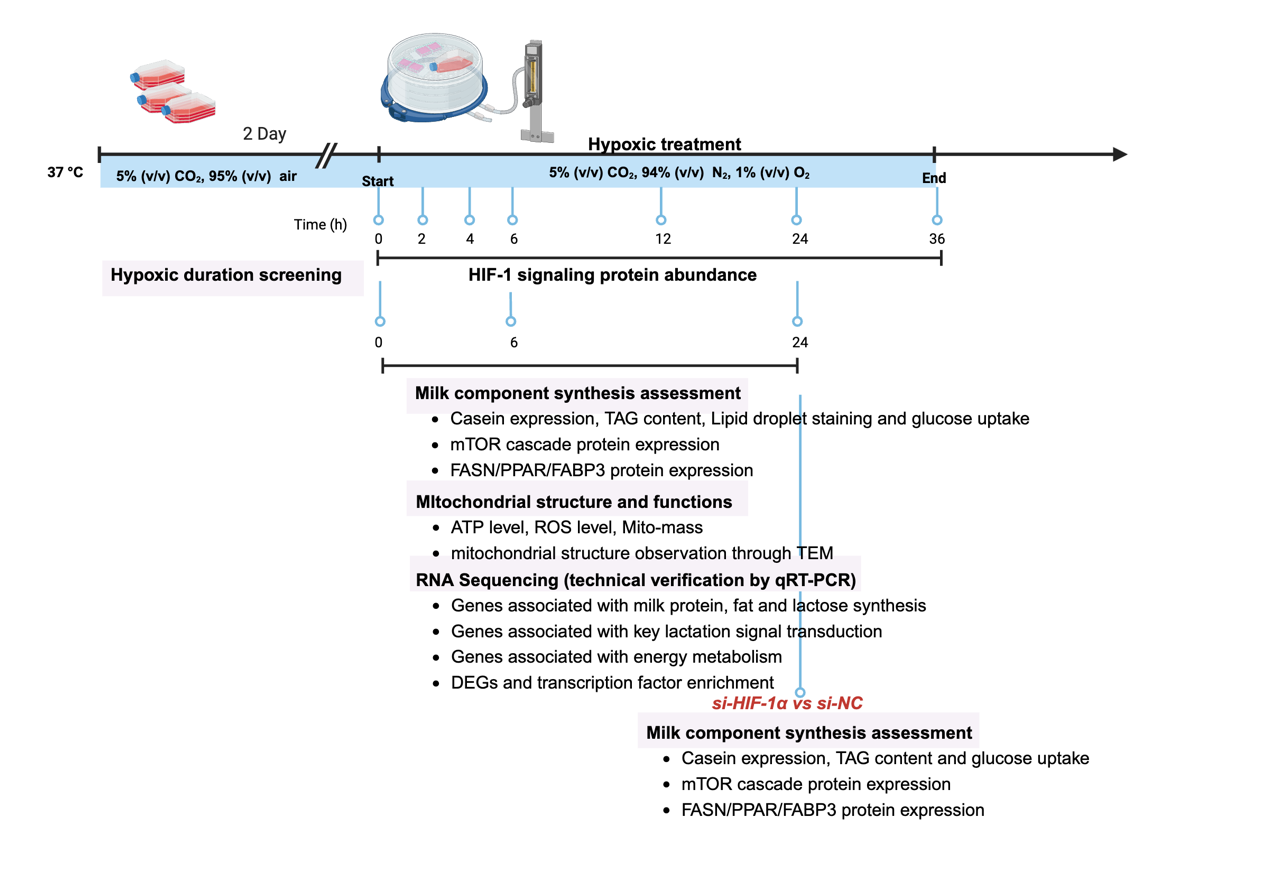


**Fig. S1. Scheme of the study workflow.**

Supplement: Supplementary file 1 — Additional file 1: Fig. S1. Scheme of the study workflow. [file 40104_2025_1174_MOESM1_ESM.docx]

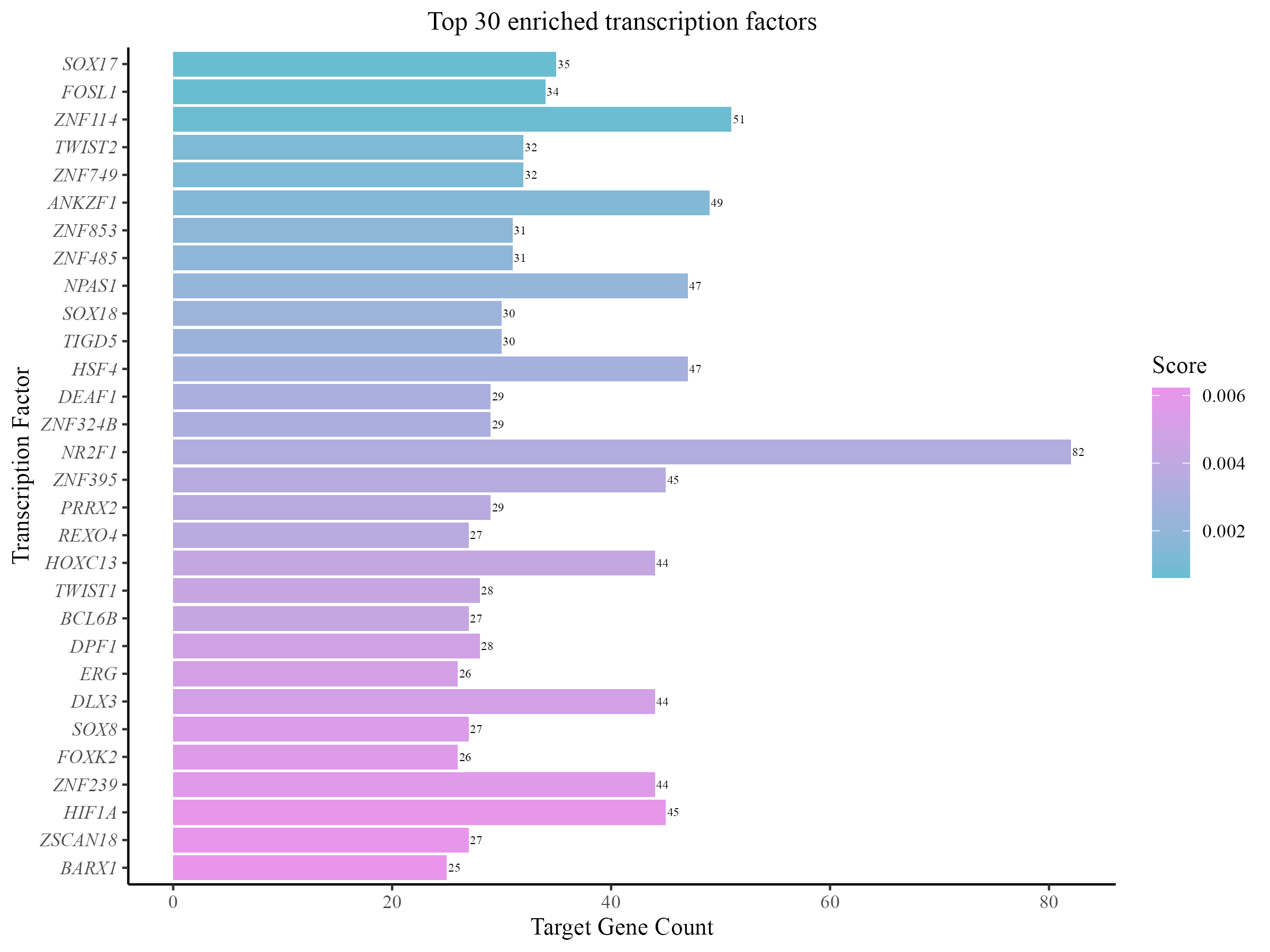


**Fig. S5. Transcription factor enrichment of DEGs in HP24 vs HP0 comparison.**

Supplement: Supplementary file 5 — Additional file 5: Fig. S5. Transcription factor enrichment of DEGs in HP24 vs. HP0 comparison. [file 40104_2025_1174_MOESM5_ESM.docx]
