# Supplementary material for: Effects of hypoxia stress on the milk synthesis in bovine mammary epithelial cells
Source: J Anim Sci Biotechnol. 2025 Mar 7;16:37. doi: 10.1186/s40104-025-01174-0 (PMC11887346; doi:10.1186/s40104-025-01174-0)
Supplement: Supplementary file 2 — Additional file 2: Fig. S2. qRT-PCR verification of DEGs from RNA sequencing. (A) Analysis of ACTB CT values across three groups (n = 4). Data with error bars represent mean ± SEM. (B) Pearson correlation analysis of the log2FC of DEGs tested by qRT-PCR and RNA sequencing. [file 40104_2025_1174_MOESM2_ESM.docx]

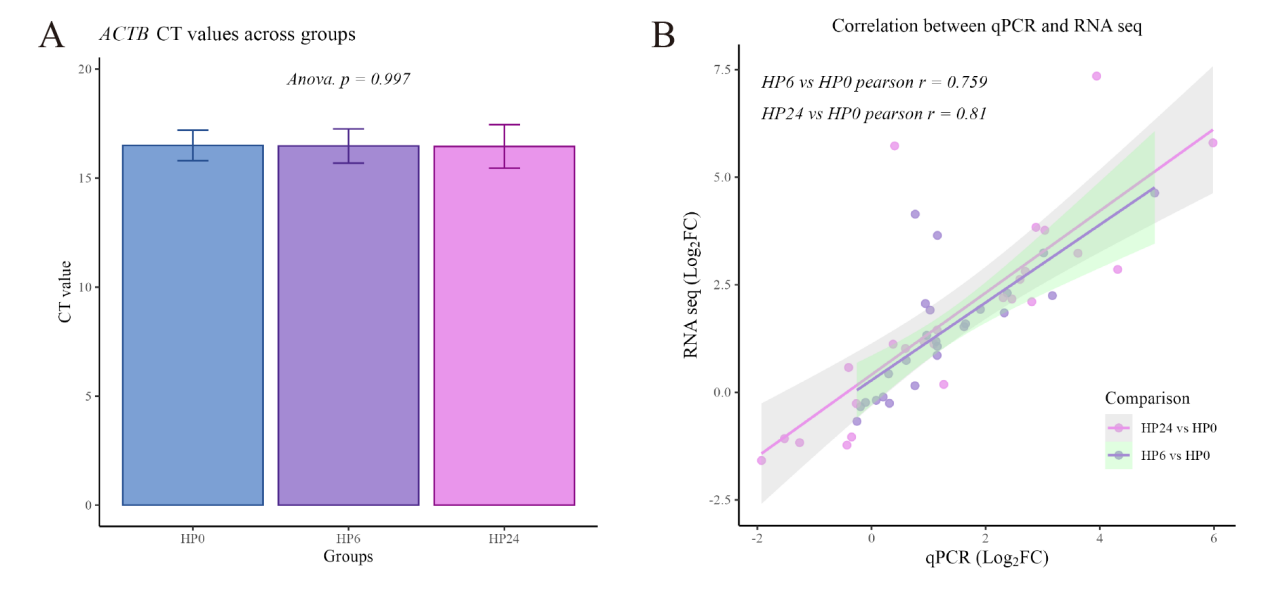


**Fig. S2. qRT-PCR verification of DEGs from RNA sequencing.** (A) Analysis of *ACTB* CT values across three groups (n = 4). Data with error bars represent mean$\text{ ± }$SEM. (B) Pearson correlation analysis of the log2FC of DEGs tested by qRT-PCR and RNA sequencing.
