# Supplementary material for: Effects of hypoxia stress on the milk synthesis in bovine mammary epithelial cells
Source: J Anim Sci Biotechnol. 2025 Mar 7;16:37. doi: 10.1186/s40104-025-01174-0 (PMC11887346; doi:10.1186/s40104-025-01174-0)
Supplement: Supplementary file 3 — Additional file 3: Fig. S3. Transcriptomic signatures of BMECs under hypoxia for 0, 6, 24 h. (A) PCA (principal component analysis) score plots. (B) Summary of the numbers of up-regulated and down-regulated differentially expressed genes (DEGs) in both hypoxia groups. (C and D) Volcano plots of genes detected in 6 h hypoxia group (HP6) (C) and 24 h hypoxia group (HP24) (D) compared to normoxia group. (E and F) Heatmap of the transcriptome in 6 h hypoxia (E) and 24 h hypoxia (F) groups compared to the normoxia group. The data underlying this figure can be found in the Table S3 and Table S4. [file 40104_2025_1174_MOESM3_ESM.docx]

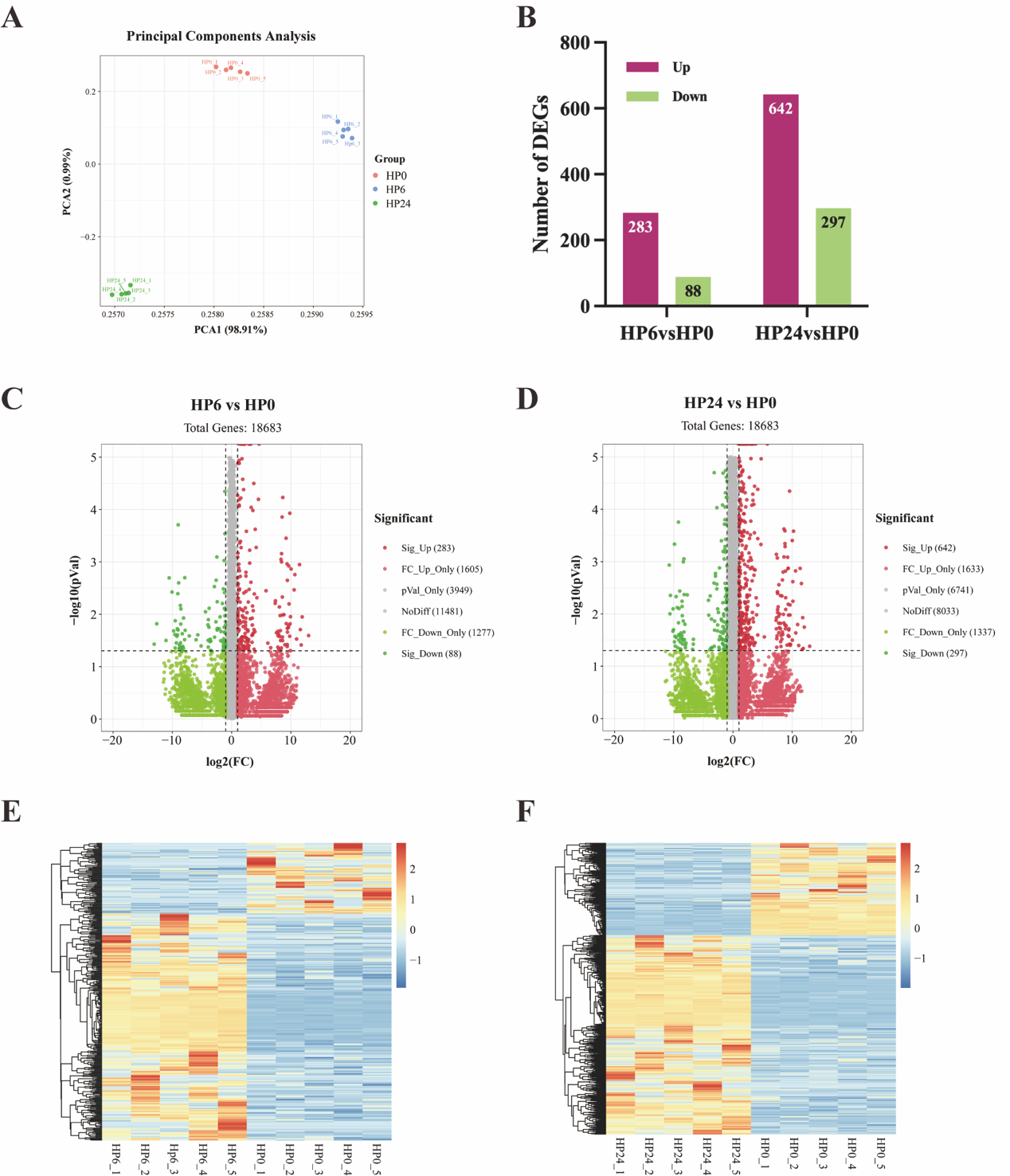


**Fig. S3. Transcriptomic signatures of BMECs under hypoxia for 0, 6, 24 h.** (A) PCA (principal component analysis) score plots. (B) Summary of the numbers of up-regulated and down-regulated differentially expressed genes (DEGs) in both hypoxia groups. (C & D) Volcano plots of genes detected in 6 h hypoxia group (HP6) (C) and 24 h hypoxia group (HP24) (D) compared to normoxia group. (E & F) Heatmap of the transcriptome in 6 h hypoxia (E) and 24 h hypoxia (F) groups compared to the normoxia group. The data underlying this figure can be found in the Table S3 and Table S4.
