# Supplementary material for: Effects of hypoxia stress on the milk synthesis in bovine mammary epithelial cells
Source: J Anim Sci Biotechnol. 2025 Mar 7;16:37. doi: 10.1186/s40104-025-01174-0 (PMC11887346; doi:10.1186/s40104-025-01174-0)
Supplement: Supplementary file 4 — Additional file 4: Fig. S4. GO enrichment of DEGs in BMECs under hypoxia. (A) and (B) shows the GO enrichment results in hypoxia for 6 h or 24 h respectively, compared to normoxia. The data underlying this figure can be found in the Table S5 and Table S6. [file 40104_2025_1174_MOESM4_ESM.docx]

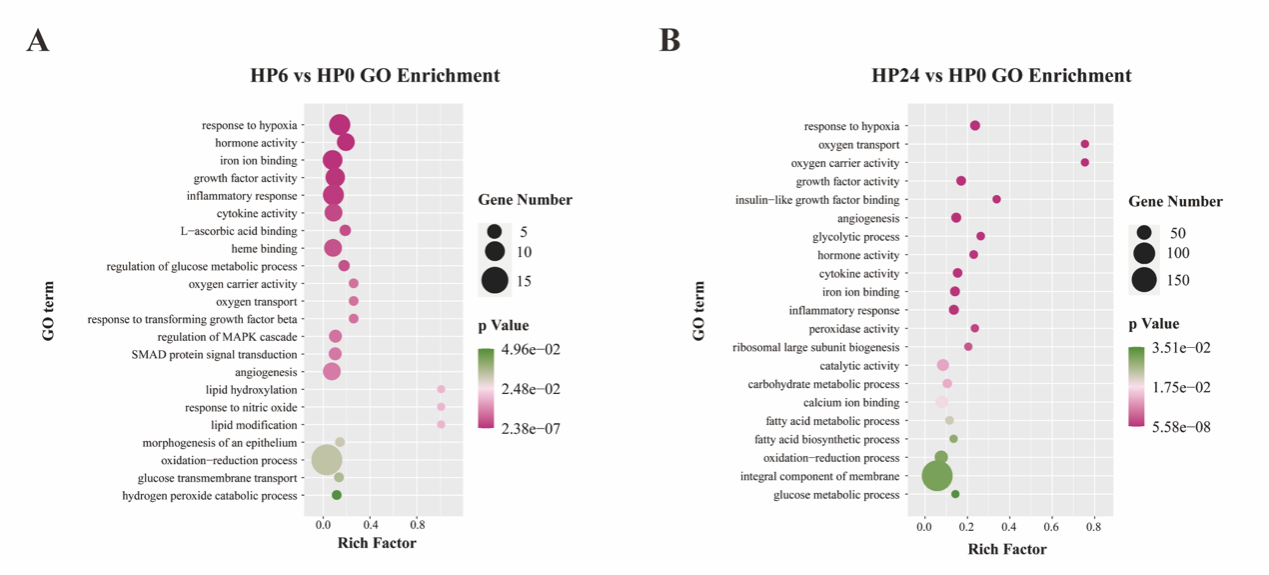


**Fig. S4. GO enrichment of DEGs in BMECs under hypoxia.** (A) and (B) shows the GO enrichment results in hypoxia for 6 h or 24 h respectively, compared to normoxia. The data underlying this figure can be found in the Table S5 and Table S6.
