# Supplementary material for: Effects of hypoxia stress on the milk synthesis in bovine mammary epithelial cells
Source: J Anim Sci Biotechnol. 2025 Mar 7;16:37. doi: 10.1186/s40104-025-01174-0 (PMC11887346; doi:10.1186/s40104-025-01174-0)
Supplement: Supplementary file 6 — Additional file 6: Fig. S6. Effects of HIF-1α knockdown on mTOR signaling proteins phosphorylation in BMECs. (A) Immunoblots of the phosphorylated forms (P) and total levels (T) of the mTOR signaling proteins in BMECs under 24 h hypoxia and transfected with either a control (NC) siRNA or HIF-1α siRNA (n = 3). (B–D) Quantitative analyses of the blot densities of the P/T forms of the mTOR signaling proteins in A. Data with error bars represent mean ± SEM. Statistical significance (P < 0.05) was determined by a two-tailed unpaired t-test. [file 40104_2025_1174_MOESM6_ESM.docx]

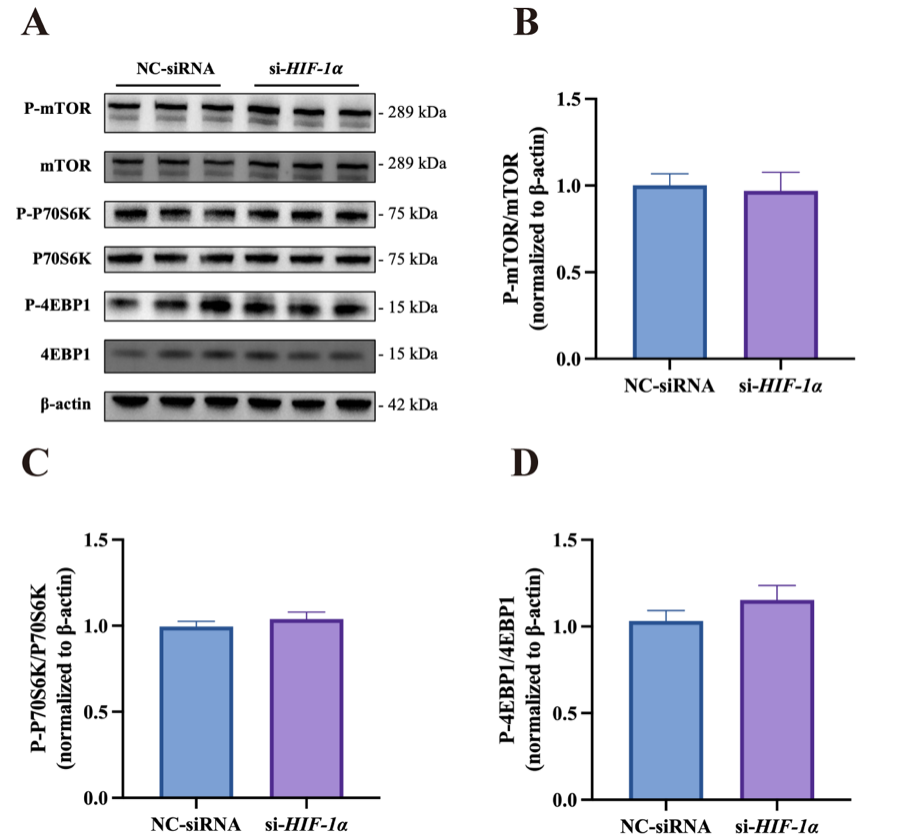


**Fig. S6. Effects of *HIF-1***$\text{α}$ **knockdown on mTOR signaling proteins phosphorylation in BMECs.** (A) Immunoblots of the phosphorylated forms (P) and total levels (T) of the mTOR signaling proteins in BMECs under 24 h hypoxia and transfected with either a control (NC) siRNA or *HIF-1*$\text{α}$ siRNA (n = 3). (B-D) Quantitative analyses of the blot densities of the P/T forms of the mTOR signaling proteins in A. Data with error bars represent mean$\text{±}$ SEM. Statistical significance (*P* < 0.05) was determined by a two-tailed unpaired *t*-test.
